# *EMSY* expression affects multiple components of the skin barrier with relevance to atopic dermatitis

**DOI:** 10.1016/j.jaci.2019.05.024

**Published:** 2019-08

**Authors:** Martina S. Elias, Sheila C. Wright, Judit Remenyi, James C. Abbott, Susan E. Bray, Christian Cole, Sharon Edwards, Marek Gierlinski, Mateusz Glok, John A. McGrath, William V. Nicholson, Lavinia Paternoster, Alan R. Prescott, Sara Ten Have, Phillip D. Whitfield, Angus I. Lamond, Sara J. Brown

**Affiliations:** a Skin Research Group, Division of Molecular and Clinical Medicine, School of Medicine, University of Dundee, Dundee, United Kingdom; b Data Analysis/Bioinformatics Group, School of Life Sciences, University of Dundee, Dundee, United Kingdom; c NHS Research Scotland Biorepository Tayside, Ninewells Hospital and Medical School, University of Dundee, Dundee, United Kingdom; d Department of Pathology, Ninewells Hospital and Medical School, Dundee, United Kingdom; e St John's Institute of Dermatology, King's College London (Guy's Campus), London, United Kingdom; f MRC Integrative Epidemiology Unit, Population Health Sciences, Bristol Medical School, University of Bristol, Bristol, United Kingdom; g Dundee Imaging Facility, School of Life Sciences, University of Dundee, Dundee, United Kingdom; h Centre for Gene Regulation and Expression, School of Life Sciences, University of Dundee, Dundee, United Kingdom; i Lipidomics Research Facility, Division of Biomedical Sciences, University of the Highlands and Islands, Inverness, United Kingdom; j Department of Dermatology, Ninewells Hospital, Dundee, United Kingdom

**Keywords:** Atopic dermatitis, atopic eczema, EMSY, filaggrin, genetics, genomics, organotypic, lipidomics, proteomics, siRNA knockdown, AD, Atopic dermatitis, DMEM, Dulbecco modified Eagle medium, EGF, Epidermal growth factor, *FLG*, Gene encoding filaggrin, GO, Gene ontology, GWAS, Genome-wide association study, Hi-C, Genome-wide chromosome conformation capture and high-throughput sequencing to identify regions of DNA showing interaction in 3-dimensional space, *LRRC32*, Leucine-rich repeat-containing 32 gene, encoding the glycoprotein A repetitions predominant (GARP) protein, NHK, Normal human keratinocytes, NDF, Normal dermal fibroblasts, qPCR, Quantitative PCR, siRNA, Small interfering RNA, SNP, Single nucleotide polymorphism, TEWL, Transepidermal water loss

## Abstract

**Background:**

Atopic dermatitis (AD) is a common, complex, and highly heritable inflammatory skin disease. Genome-wide association studies offer opportunities to identify molecular targets for drug development. A risk locus on chromosome 11q13.5 lies between 2 candidate genes, *EMSY* and *LRRC32* (leucine-rich repeat-containing 32) but the functional mechanisms affecting risk of AD remain unclear.

**Objectives:**

We sought to apply a combination of genomic and molecular analytic techniques to investigate which genes are responsible for genetic risk at this locus and to define mechanisms contributing to atopic skin disease.

**Methods:**

We used interrogation of available genomic and chromosome conformation data in keratinocytes, small interfering RNA (siRNA)–mediated knockdown in skin organotypic culture and functional assessment of barrier parameters, mass spectrometric global proteomic analysis and quantitative lipid analysis, electron microscopy of organotypic skin, and immunohistochemistry of human skin samples.

**Results:**

Genomic data indicate active promoters in the genome-wide association study locus and upstream of *EMSY*; *EMSY*, *LRRC32*, and intergenic variants all appear to be within a single topologically associating domain*.* siRNA-knockdown of *EMSY* in organotypic culture leads to enhanced development of barrier function, reflecting increased expression of structural and functional proteins, including filaggrin and filaggrin-2, as well as long-chain ceramides. Conversely, overexpression of *EMSY* in keratinocytes leads to a reduction in markers of barrier formation. Skin biopsy samples from patients with AD show greater EMSY staining in the nucleus, which is consistent with an increased functional effect of this transcriptional control protein.

**Conclusion:**

Our findings demonstrate an important role for *EMSY* in transcriptional regulation and skin barrier formation, supporting EMSY inhibition as a therapeutic approach.

## Graphical abstract

**Figure:**
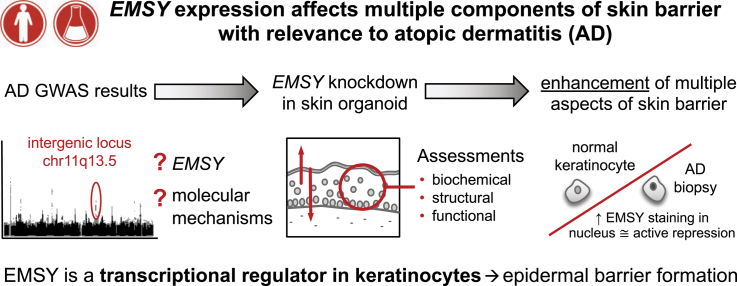

Atopic dermatitis (AD; or eczema^1^) is a common inflammatory skin disease with strong heritability.^2^ Genome-wide association studies (GWASs) have identified multiple loci affecting AD risk,^3^ including effects on the skin barrier and immune function,^2^, ^4^ and it has been demonstrated in other complex traits that molecular mechanisms defined by GWAS loci might represent effective therapeutic targets.^5^

The most widely replicated genetic risk for AD lies within the epidermal differentiation complex on chromosome 1q21.3^3^, ^6^, ^7^; this includes *FLG*, which encodes the skin barrier protein filaggrin.^8^ Expression levels of filaggrin and its metabolites in the outer epidermis correlate with AD activity.^9^, ^10^ However, this mechanism has not been successfully targeted in therapy development since its discovery more than 10 years ago.

An association peak within an intergenic region on chromosome 11q13.5 was identified in the earliest AD GWAS.^6^ This locus has been replicated in subsequent GWASs^11^, ^12^, ^13^ and meta-GWASs.^3^, ^7^ In addition to AD, the region is associated with multiple atopic phenotypes^14^, ^15^ and other disorders characterized by epithelial barrier dysfunction, including polyallergen sensitization,^14^, ^16^ asthma,^17^ allergic rhinitis,^18^ food allergy,^19^, ^20^ eosinophil counts,^21^ eosinophilic esophagitis,^22^ inflammatory bowel disease,^23^ and the gut microbiome.^24^ The AD-associated single nucleotide polymorphisms (SNPs) are in an intergenic region between *LRRC32* (leucine-rich repeat-containing 32), and *EMSY*.

*LRRC32* encodes the TGF-β activator LRRC32 (UniProtKB Q14392), previously termed glycoprotein A repititions predominant (GARP), a membrane protein that binds latent TGF-β1 on the surfaces of activated regulatory T cells.^25^ *LRRC32* has been proposed as a causal gene for atopic skin inflammation,^26^ but the credible SNPs identified by GWASs at this locus are all intergenic,^3^ suggesting that regulatory rather than coding variants drive the association.

*EMSY*, also known as BRCA2-interacting transcriptional repressor and previously termed *C11orf30*, codes for the protein EMSY (UniProtKB Q7Z589), which is expressed in multiple human tissues, including cerebellum, breast, lung, ovary, uterus, and skin (GTEx RNA-seq, V7). EMSY has been characterized as a transcriptional regulator, either repressing transcription as part of a chromatin remodeling complex or activating transcription as part of a histone H3–specific methyltransferase complex.^27^ *EMSY* amplification is associated with DNA damage and increased risk of malignancy in breast and ovarian tissue.^27^ EMSY can also play a role in inflammation because the protein kinase AKT1 regulates the interferon response through phosphorylation of EMSY,^28^ but its role in skin remains undefined.

We set out to investigate, using genetic and genomic data, whether *EMSY*, *LRRC32*, or both, showed evidence of activity in human skin cells. We then investigated the mechanism of effect using functional and multi-omics analysis of organotypic skin, followed by immunostaining of AD biopsy samples.

## Methods

### Human tissues and cells

All human tissues were obtained with written informed consent from donors under the governance of and with ethical approval from the NHS Research Scotland Biorepository in Tayside. Primary keratinocytes and donor-matched primary fibroblasts were extracted from skin samples discarded from plastic surgery procedures. AD skin samples were identified from the hospital pathology database as consecutive unselected cases. Demographic details are provided in the Methods section in this article's Online Repository at www.jacionline.org.

### Cell and skin organotypic cultures

Normal human keratinocytes (NHKs) and normal dermal fibroblasts (NDFs) were isolated from healthy breast skin by means of sequential collagenase D and trypsin EDTA digestion, as previously described.^29^ NHKs were cocultured in RM medium (3:1 Dulbecco modified Eagle medium [DMEM]/Hams F12, 10% FCS, 0.4 μg/mL hydrocortisone, 5 μg/mL insulin, 10 ng/mL epidermal growth factor [EGF], 5 μg/mL transferrin, 8.4 ng/mL cholera toxin, and 13 ng/mL liothyronine; Sigma-Aldrich, Gillingham, Dorset, United Kingdom) along with mitomycin C–inactivated 3T3 feeder cells.^30^ EGF was omitted for the first day of culture. NDFs were cultured in DMEM supplemented with 10% FCS under standard conditions.

Fibrin gel dermal equivalents were prepared by using a protocol adapted from published methods.^31^, ^32^, ^33^ A volume of 0.5 mL fibrinogen (35 mg/mL in NaCl; Sigma-Aldrich) and 0.5 mL of thrombin (3 U/mL in 2 mmol/L CaCl_2_/1.1% NaCl; Sigma-Aldrich) were combined on ice, supplemented with 200,000 NDFs and aprotinin (0.1 U/mL; Sigma-Aldrich), and transferred to a 12-well plate. After 30 minutes of incubation at 37°C, the gels were covered in medium (DMEM, 10% FCS, and 0.1 U/mL aprotinin) and cultured overnight (day 1). The following day, medium was replaced with RM medium excluding EGF, 0.1 U/mL aprotinin, and 2 × 10^6^ suspended NHKs (day 2). This was refreshed daily for the next 2 days (day 3 and 4) with RM medium containing 0.1 ng/mL EGF and 0.1 U/mL aprotinin. On day 5, gels were carefully removed from wells by using a plastic spatula and lifted onto custom-made steel grids lined with nylon gauze (Millipore, Livingston, United Kingdom). RM medium supplemented with 0.1 ng/mL EGF and 0.1 U/mL aprotinin was added up to the base of the grid, enabling the fibrin gels to be nourished from below and the epidermis cultured at the air-liquid interface. Medium was refreshed every other day until day 12 when the cultures were analyzed. Where required, the epidermis was isolated from fibrin gel after hypertonic saline–induced split (4 hours, 1 mol/L NaCl, 4°C).

### Small interfering RNA–mediated knockdown

NHKs were reverse transfected immediately before inclusion in the organotypic cultures by using RNAiMax transfection reagent (Life Technologies, Carlsbad, Calif), according to the manufacturer's instructions. Briefly, small interfering RNA (siRNA) complexes were formed in Opti-MEM medium (20 μmol/L siRNA and 5 μL of RNAiMAX) and, after 20 minutes of incubation, combined with 2 × 10^6^ suspended NHKs and transferred to the preprepared dermal substrate. A pool of 4 siRNA duplexes was used (EMSY: LQ-004081-00-0002, FLG: LQ-021718-00-0002, control: ON-TARGETplus nontargeting siRNA #4 D.001810-04-20; Dharmacon, Lafayette, Colo).

### *EMSY* overexpression in primary keratinocytes

A second-generation lentiviral system was used, as follows. Pseudoviral particles were prepared with psPAX2 packaging (catalog no. 12260; AddGene, Watertown, Mass) and pMD2.G plasmid (catalog no. 12259; AddGene), which were cotransfected with the control pLenti-C-mGFP-P2A-puromycin–tagged cloning vector (catalog no. PS100093; OriGene, Rockville, Md) plasmid or with the Lenti-ORF clone of mGFP-tagged-human chromosome 11 open reading frame 30 (*C11orf30*; catalog no. RC216916L4; OriGene) plasmid with Lipofectamine-3000 transfection reagent (catalog no. L3000008; Invitrogen, Carlsbad, Calif), according to the manufacturer's protocol, into 293T packaging cells for 16 hours. The next morning, the cells were washed twice with PBS to remove excess plasmid DNA, and the medium was replaced with virus-producing medium (20% FBS/DMEM). Forty-eight, 72, and 96 hours after transfection, the first, second, and third viral supernatants were harvested. Viral supernatants were spun down at 1200 rpm for 15 minutes and filtered with a 0.45-μm filter.

Primary human keratinocytes from donor skin were transduced twice. The first transduction was in RM medium without EGF. After treatment, the keratinocytes with trypsin and 1 × 10^6^ cells/well were mixed with 10 μg/mL Polybrene (hexadimethrine bromide; catalog no. H9268; Sigma-Aldrich) and 1 or 2 mL of viral supernatant and then plated onto 6-well plates and cultured overnight. The second transduction was performed in monolayer culture using RM medium without EGF, 10 μg/mL Polybrene, and 1 or 2 mL of viral supernatant. Cells were incubated for 90 minutes at 37°C, followed by centrifugation at 1200 rpm. To eliminate excess virus, cells were washed twice with PBS, and the medium was replaced with RM media (3:1 DMEM/Hams F12, 10% FCS, 0.4 μg/mL hydrocortisone, 5 μg/mL insulin, 10 ng/mL EGF, 5 μg/mL transferrin, 8.4 ng/mL cholera toxin, and 13 ng/mL liothyronine). Fresh RM medium was replaced every second day, and samples were harvested on day 10 after transduction as a differentiated culture.

### Fluorescent dye penetration

Fifty microliters of 1 mmol/L Lucifer yellow dye (Sigma-Aldrich) was added to the epidermal surface of the organotypic culture and incubated at 37°C for 4 hours. Metal cloning rings were used to control uniform dosing on the epidermal surface. Lucifer yellow was removed, and the organotypic cultures were washed in PBS before formalin-fixed paraffin embedding under standard conditions. Four-micrometer sections were deparaffinized, counterstained with 4′-6-diamidino-2-phenylindole dihydrochloride (1 μg/mL for 10 minutes; Life Technologies, Carlsbad, Calif), and imaged with a confocal Zeiss LSM710 microscope (Zeiss, Oberkochen, Germany). Quantification of dye penetration in the upper dermis (average intensity in the upper 40 μm) was performed with Zeiss Zen software and compared by using paired *t* tests.

### Transepidermal water loss

Organotypic cultures were equilibrated at room temperature and atmospheric conditions for 30 minutes before transepidermal water loss (TEWL) was measured at 2 locations on the epidermal surface with an AquaFlux AF200 instrument (Biox Systems, London, United Kingdom) with a custom (5 mm in diameter) probe head. TEWL measurements were taken every second for a minimum of 60 seconds until a stable reading, as determined by using the software, was obtained.

### Capacitance

Organotypic cultures were equilibrated at room temperature and atmospheric conditions for 30 minutes before measurement of epidermal surface capacitance as a measure of water content with a Corneometer (Courage and Khazaka, Cologne, Germany). Three measurements were recorded from each organotypic culture, and the mean was calculated.

### Protein data analysis

Network analysis was performed by using Ingenuity Pathway Analysis (Qiagen Ingenuity, version 01-12; Qiagen, Hilden, Germany). Gene ontology (GO) enrichment analysis was performed in the Gene Ontology Consortium online tool by using PANTHER classification http://www.geneontology.org/page/go-enrichment-analysis (accessed August 28, 2018). A volcano plot was generated in R/ggplot2 by using human proteins detected in all 4 of the replicates to calculate the fold change (nontargeting control/EMSY knockdown) for each donor with log_10_ transformation and a *t* test for significance.

### Lipid staining

Frozen sections of skin organotypic samples were cut and air-dried onto slides before formalin fixation and rinsing with 60% isopropranol. Oil Red O (Sigma-Aldrich) working solution was freshly prepared, and sections were stained for 15 minutes, rinsed with 60% isopropanol, and then lightly counterstained with alum hematoxylin before a final rinse with distilled water.

Additional Methods are described in this article's Online Repository at www.jacionline.org.

## Results

### Genomic data support *EMSY* and *LRRC32* as candidate genes in skin

**Fig 1:**
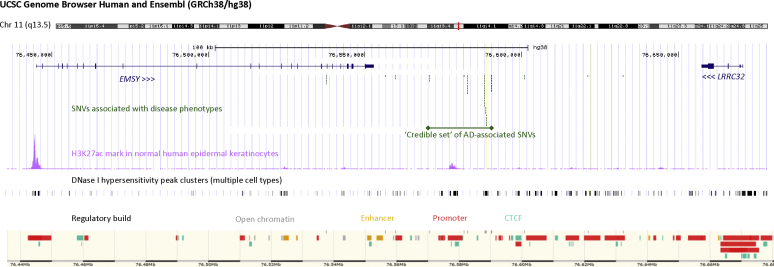
Regulatory features of the AD risk locus and adjacent genes on chromosome 11q13.5. Genes *(blue)* flanking the locus with multiple disease-associated SNPs *(green)* were identified by using a GWAS. *EMSY* has a protein-coding region spanning 106 kb, with 20 exons producing 18 splice variants. *LRRC32* spans 13 kb, including 3 exons. The location of the credible set of AD-associated SNPs^3^ is marked. H3K27ac marks *(pink)* and DNase I hypersensitivity data *(gray)* are from the Encyclopedia of DNA Elements (ENCODE; accessed June 2018). Regulatory features in the same region of chromosome 11 are from Ensembl (GRCh38.12, accessed April 2019).

The AD-associated SNPs at the chromosome 11q13.5 locus are approximately 27 kb downstream of *EMSY* (Human Genome Nomenclature Committee: 18071, ENSG00000158636) and approximately 77 kb downstream of *LRRC32* (Human Genome Nomenclature Committee: 137207, ENSG00000137507). The credible set identified by mapping this locus lies entirely within an intergenic region (Fig 1). Encyclopedia of DNA Elements (ENCODE) and Ensembl data predict multiple regulatory features within the region of disease risk SNPs, and there are putative promoters upstream of each gene (Fig 1). Focusing on skin, there are histone H3K27ac marks indicating active enhancers or promoters in NHKs in the AD risk locus and at the 3′ end of *EMSY* but not *LRRC32* (Fig 1).

Enhancer-promoter interactions can occur by proximity in 3-dimensional space^34^ and show cell lineage specificity^35^; therefore we reanalyzed 2 sets of genome-wide chromosome conformation capture and high-throughput sequencing (Hi-C) data from NHKs, to identify regions of DNA showing interaction in 3-dimensional space. Interrogation of Hi-C data^36^ shows that the intergenic SNPs, as well as *EMSY* and *LRRC32* all lie within a single topologically associated domain in keratinocytes (see Fig E1 in this article's Online Repository at www.jacionline.org), supporting a possible functional interaction. Analysis of promoter-capture Hi-C^37^ in differentiating keratinocytes showed evidence of interaction between the promoter region of *LRRC32* and the intergenic SNP locus, but these data were not sufficiently detailed to determine whether *EMSY* also shows conformational interaction (see Fig E1).

Gene expression data^38^ and our own single-molecule RNA-sequencing analysis^39^ confirm expression of each gene in the skin, but there is no significant difference in *EMSY* or *LRRC32* mRNA abundance in atopic skin compared with nonatopic control skin (*P* > .05, see Fig E2 in this article's Online Repository at www.jacionline.org). However, *EMSY* is more highly expressed in skin than *LRRC32* at the protein level (https://www.proteinatlas.org/), and it has not previously been studied in keratinocyte biology; therefore *EMSY* was selected for further detailed investigation.

### *EMSY* knockdown in a skin organotypic model enhances barrier function

**Fig 2:**
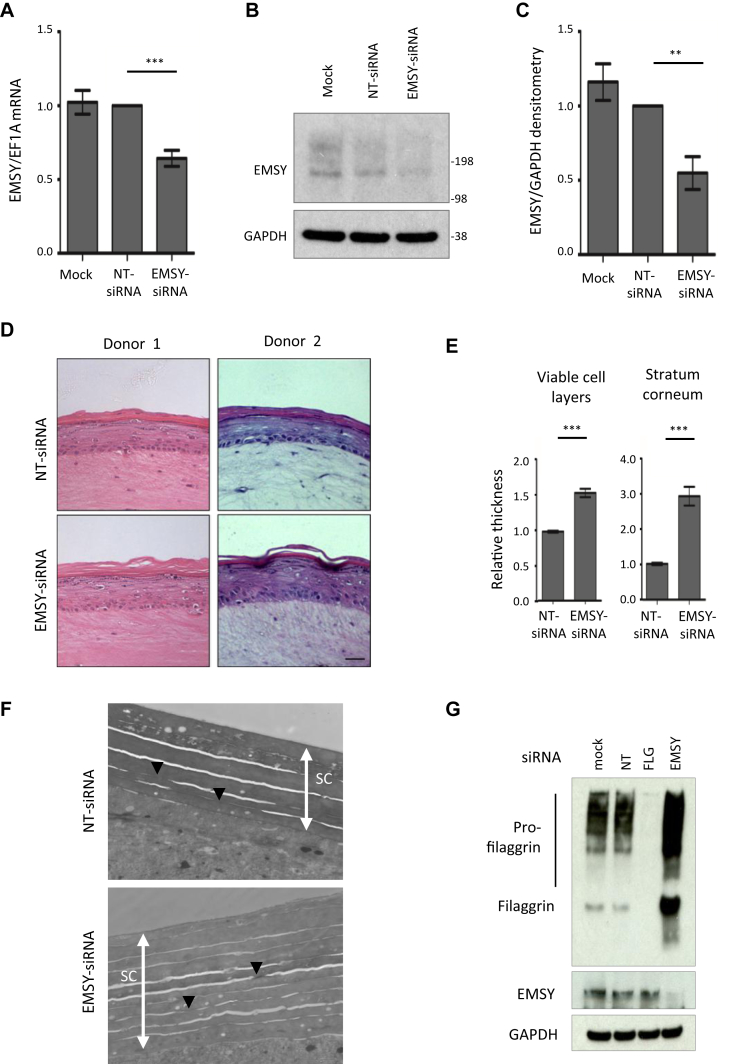
Biochemical and histologic effects of *EMSY* knockdown in a skin organotypic model *in vitro*. **A,** qPCR showing knockdown of *EMSY* mRNA after 10 days in culture (7 days after lifting to the air-liquid interface) normalized to a nontargeting control–treated sample (n = 7, mean 36% reduction compared with the nontargeting control). **B** and **C,** Western blot showing knockdown of EMSY protein after 10 days in culture (Fig 2, *B*) and densitometry to quantify protein knockdown normalized to a nontargeting control (Fig 2, *C*; n = 7). **D,** Appearance of organotypic cultures showing an increased granular layer and thickened stratum corneum in response to *EMSY* siRNA knockdown; representative images were replicated in 10 independent donor experiments. *Scale bar* = 25 μm. **E,** Relative thickness of epidermal layers (n = 10). **F,** Transmission electron microscopy of stratum corneum showing thickness of the stratum corneum *(white arrows)* with an increase in the number of layers and number of corneodesmosomes *(black arrowheads)*. **G,** Western blot showing filaggrin expression in skin organotypic cultures untreated (mock) and treated with nontargeting siRNA, *FLG* siRNA, and *EMSY* siRNA 7 days after lifting to the air-liquid interface. **P < .001 and ***P < .0001, paired *t* test. *GAPDH*, Glyceraldehyde-3-phosphate dehydrogenase; *NT*, nontargeting control siRNA. *Bars* show means and SEMs.

To investigate a functional effect of EMSY in skin, we used primary human keratinocytes seeded onto a dermal equivalent, which forms an organotypic model with stratified layers that effectively recapitulate the structure and gene expression patterns of human skin.^40^ The model also demonstrates functional parameters controlling the entry and exit of small molecules, and this can be used to quantify effects on barrier formation and function.^41^ siRNA knockdown of *EMSY* expression was achieved by means of transfection of keratinocytes immediately before seeding onto the dermal equivalent. Knockdown was confirmed at the mRNA and protein levels, persisting to 10 days in organotypic culture (Fig 2, *A-C*). Equivalent effects were seen by using individual and pooled siRNAs (see Fig E3 in this article's Online Repository at www.jacionline.org). *EMSY* knockdown produced a marked phenotypic change (Fig 2, *D*), including thickening of the epidermal cell layer and stratum corneum (Fig 2, *E*). There was an increase in the number of layers within the stratum corneum and the frequency of corneodesmosomes (Fig 2, *F*). The stratum granulosum, the site of filaggrin expression,^8^ was also more prominent (Fig 2, *D*), and increased filaggrin expression was confirmed by using quantitative PCR (qPCR; n = 7 replicates, mean ± SEM fold change = 2.00 ± 0.41 compared with the nontargeting control) and Western blotting (n = 7, mean fold change = 1.97 ± 0.22; Fig 2, *G*).

**Fig 3:**
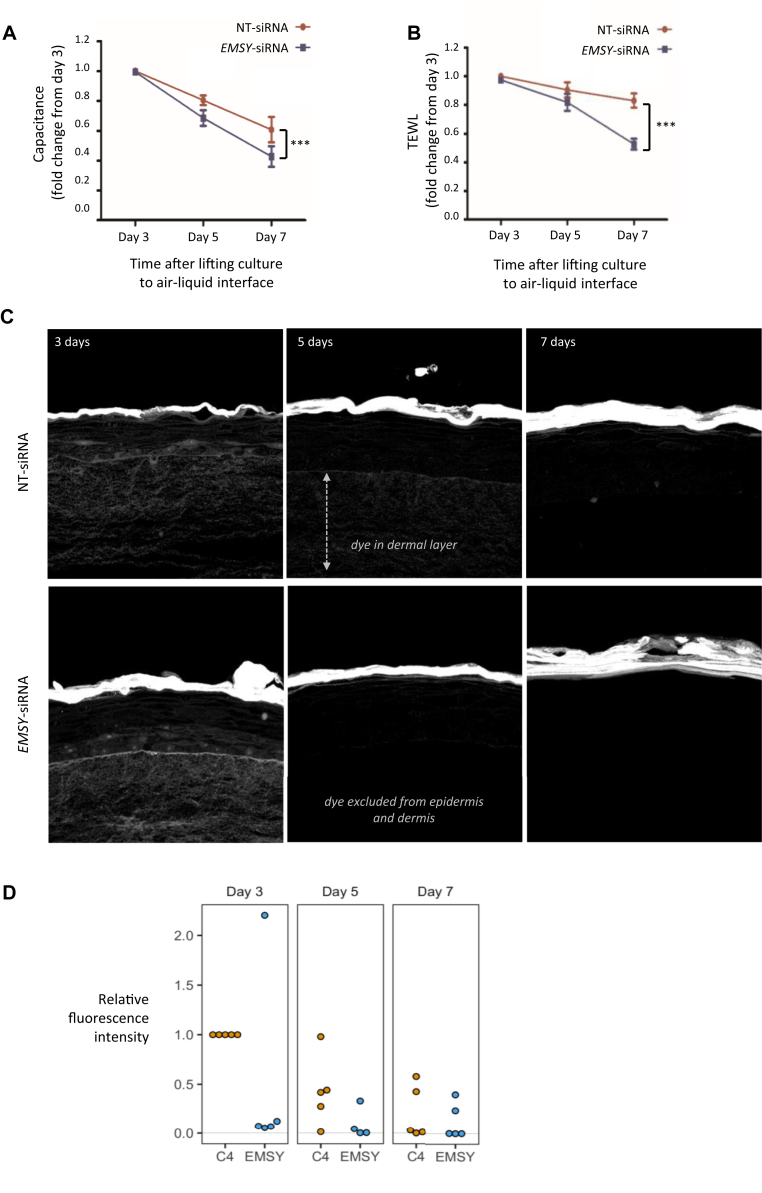
Functional effects of *EMSY* knockdown in the skin organotypic model. **A,** Water content of the stratum corneum measured by capacitance on the skin surface (n = 5). ***P < .0001, paired *t* test. **B,** Water evaporation from the epidermal surface measured as TEWL (n = 5). **C,** Visualization of fluorescent dye penetration through the epidermis and dermis after 4 hours in skin organotypic cultures developed for 3, 5, and 7 days after lifting to the air-liquid interface. Representative images are from experiments replicated in 5 independent donor experiments. **D,** Overexpression of *EMSY* in primary human keratinocytes and concomitant reduction in expression of differentiation markers (n = 5).

In our skin culture model stratum corneum hydration, TEWL, and Lucifer yellow dye penetration progressively decrease as the skin barrier is formed (see Fig E4 in this article's Online Repository at www.jacionline.org). *EMSY* knockdown in the skin model resulted in a reduction in stratum corneum hydration (Fig 3, *A*), a reduction in TEWL (Fig 3, *B*), and a reduction in penetration of the Lucifer yellow dye (Fig 3, *C* and *D*), which is in keeping with enhanced and accelerated barrier development compared with control siRNA treatment.

### *EMSY* overexpression reduces filaggrin expression

**Fig 4:**
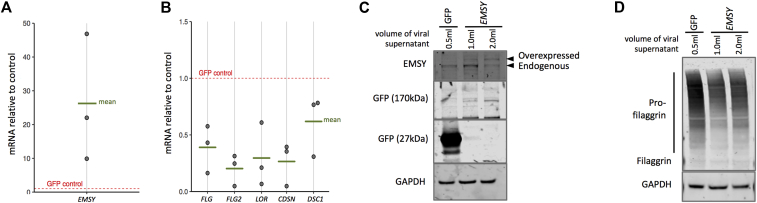
Functional effects of *EMSY* knockdown in a skin organotypic model. Overexpression of *EMSY* in primary human keratinocytes **(A)** and concomitant reduction in expression of differentiation markers at the mRNA **(B)** and protein (**C** and **D**) levels (n = 3). Quantification of protein blots by using densitometry is shown in Fig E5. *GAPDH*, Glyceraldehyde-3-phosphate dehydrogenase; *GFP*, green fluorescent protein.

In contrast to the phenotype observed with *EMSY* knockdown, overexpression of *EMSY* in primary human keratinocytes resulted in a reduction in multiple markers of differentiation and barrier formation at the mRNA and protein levels (Fig 4 and see Fig E5 in this article's Online Repository at www.jacionline.org).

### Proteomic analysis reveals pathways inhibiting the development of dermatitis

**Fig 5:**
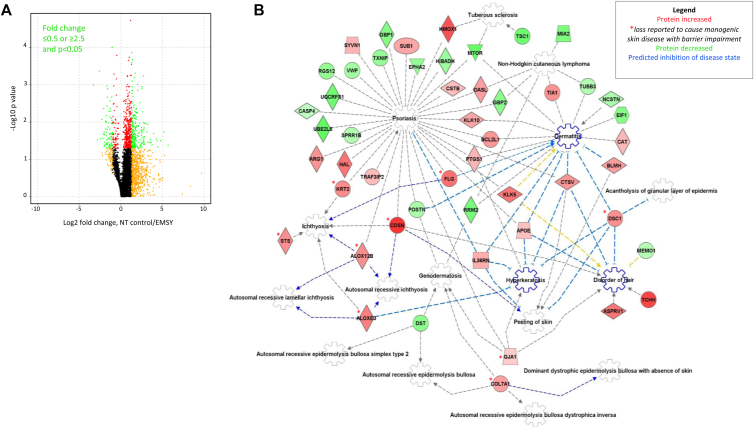
Proteomic analysis of *EMSY* knockdown in a skin organotypic model. **A,** Volcano plot showing mean fold change in 4 biological replicate samples comparing the nontargeting *(NT)* control siRNA–treated model with *EMSY* siRNA knockdown. *t* Test results are color coded red (*P* < .05), orange (fold change ≤ 2.5 or ≥ 0.5), or green (*P* < .05 and fold change ≥ 2.5 or ≤ 0.5). **B,** Ingenuity Pathway Analysis (Qiagen) of proteins consistently upregulated or downregulated (0.5 ≥ fold change ≥ 2.5) in 3 or more of 4 biological replicates, showing proteins defective in monogenic skin diseases with similarities to AD, including ichthyoses,^42^, ^43^, ^44^, ^45^ hyperkeratosis,^46^ or skin fragility^47^, ^48^*(red asterisks)* and an enhancement of pathways predicted to inhibit the development of dermatitis, hyperkeratosis, and hair disorders *(blue cogwheels)*.

To assess in more detail the *EMSY* knockdown phenotype, we applied tandem mass spectrometric global proteomic analysis (MS/MS) to donor-matched control and EMSY knockdown organotypic experiments from 4 independent donors. Total epidermal protein extracts were fractionated by using high-pH reversed-phase chromatography before tandem mass spectrometry, which identified more than 9000 proteins per sample (see Fig E6 in this article's Online Repository at www.jacionline.org). qPCR and Western blotting confirmed knockdown of EMSY in each sample. A greater proportion of proteins showed increased rather than decreased expression on *EMSY* knockdown (Fig 5, *A*), indicating that EMSY's predominant role is as a transcriptional repressor in this tissue.

Data were filtered for proteins showing changes in the same direction consistently across all 4 biological replicates and fold changes of 2.5 or greater (n = 154 proteins) or 0.5 or less (n = 130 proteins) in at least 3 of 4 of the biological replicates. Consistent direction of change was used as a criterion to focus analysis on changes of relevance to *EMSY* knockdown and showing reproducibility across different donors to minimize any effect of interindividual differences.

GO analysis of the upregulated proteins showed enrichment for the biological processes termed establishment of the skin barrier (GO: 0061436), skin development (GO: 0043588), regulation of water loss through the skin (GO: 0033561), and cornification (GO: 0070268; each false discovery rate: *P* ≤ .037). GO analysis of the downregulated proteins showed enrichment of cellular components termed cytosol (GO: 0005829), the mitochondrial membrane (GO: 0031966), and the Golgi membrane (GO: 0000139; each false discovery rate: *P* ≤ .010). The full lists of GO terms defined by using this analysis are shown in Fig E7 in this article's Online Repository at www.jacionline.org.

Pathway analysis (Ingenuity, version 01-12; Qiagen) identified upregulation of pathways predicted to inhibit the development of dermatitis and ichthyosis (Fig 5, *B*).^42^, ^43^, ^44^, ^45^, ^46^, ^47^, ^48^ Differential expression levels of proteins from these pathways were tested for validation by using qPCR, Western blotting, and/or immunostaining of organotypic skin. All positive and negative findings and untested proteins are displayed in Fig E8 in this article's Online Repository at www.jacionline.org.

### *EMSY* knockdown increases long-chain ceramides

**Fig 6:**
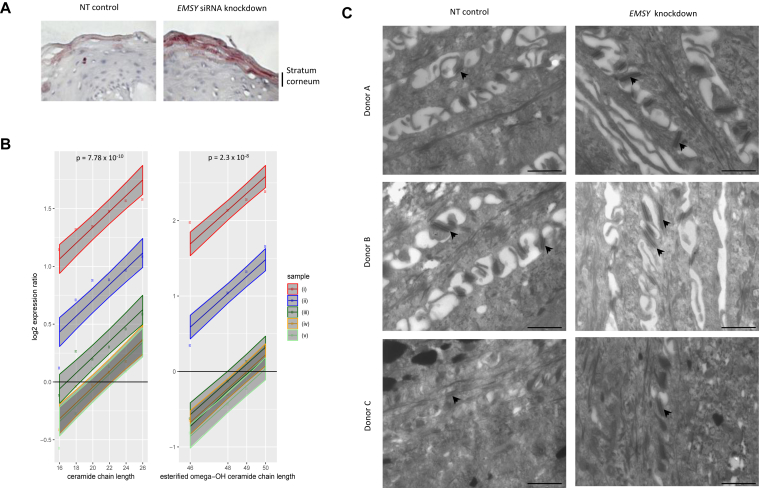
Lipid and ultrastructural analyses of EMSY knockdown in the skin organotypic model. **A,** Lipid staining of skin organotypic samples treated with nontargeting *(NT)* siRNA control and *EMSY* siRNA and Oil Red O stain neutral lipids **B,** Mass spectrometric lipid analysis showing a greater abundance of ceramides with longer chain length in *EMSY* siRNA–treated organotypic skin compared with a nontargeting siRNA control. The *x-axis* shows nonhydroxy fatty acid carbon chain length (n = 5 biological replicate samples). *Lines* show the best fit of the linear model and 95% CIs *(shaded areas)*. *P* values are for the term in the linear model for lipid chain length. **C,** Transmission electron microscopy: osmium tetroxide postfixed and stained, showing increased size of desmosomal structures *(arrows)* in the *EMSY* knockdown skin model. *Scale bar* = 1 μm.

Organotypic samples showed an increase in abundance of epidermal lipid staining with EMSY knockdown (Fig 6, *A*), which is in keeping with the increase in levels of proteins involved with lipid metabolism (eg, STS, ALOXE3, ALOX12B, and APOE). Mass spectrometric lipid analysis showed an increase in long-chain ceramides and esterified omega-hydroxy-ceramides species in organotypic samples with *EMSY* knockdown (Fig 6, *B*); these are the ceramides that have previously been reported to show a reduction in AD skin samples.^49^

### *EMSY* knockdown increases cell-cell adhesion structures

Ultrastructural analysis demonstrated an increase in desmosome size (Fig 6, *C*), which is consistent with the observed increase in desmocollin 1 levels. An increase in the number of layers within the stratum corneum was also observed, which is consistent with the increase in corneodesmosin levels (Fig 2, *F*, and see Fig E9 in this article's Online Repository at www.jacionline.org).

### EMSY shows predominantly nuclear localization in the skin affected by AD

**Fig 7:**
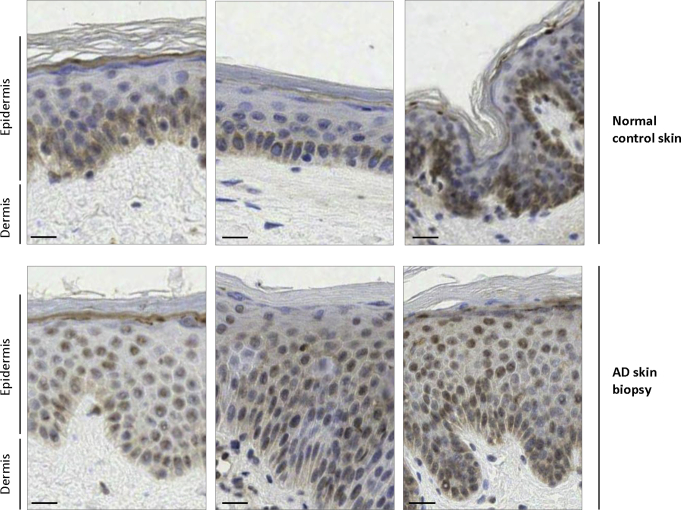
EMSY localization in skin of patients with and without AD. Immunohistochemistry showing EMSY staining in a predominantly basal distribution in normal human skin in contrast to nuclear staining extending throughout the epidermis in AD lesions. Representative images are taken from a tiled image of the whole specimen viewed by using Deep Zoom. *Scale bar* = approximately 20 μm.

To investigate *EMSY* expression in human skin samples, we compared 18 normal control skin samples and biopsy specimens from 14 patients with spongiotic dermatitis, AD, or both. Immunohistochemistry showed considerable interindividual variability in EMSY staining, despite careful standardization of staining conditions, and there was no consistent difference in total intensity between cases and control subjects (see Fig E10 in this article's Online Repository at www.jacionline.org). However, all 14 AD cases showed greater nuclear than cytoplasmic staining, and 12 (86%) of 14 cases showed predominantly nuclear staining extending throughout the epidermis compared with 2 (11%) of 18 control subjects (example images are shown in Fig 7, and all images are shown in Fig E10). Similarly, control organotypic samples showed EMSY staining in nuclei throughout the epidermis, whereas the organotypic cultures with *EMSY* knockdown showed expression predominantly in the basal epidermis in keratinocyte nuclei. Thus increased nuclear expression is consistent with increased activity of EMSY as a transcriptional regulator within keratinocytes in lesional skin of patients with AD.

## Discussion

GWASs have provided valuable insight into the pathogenic mechanisms of many common complex traits, and some have been exploited for targeted therapy development.^5^, ^50^, ^51^ However, many of the loci identified by GWASs are intergenic, and the molecular mechanisms require detailed functional analysis performed in disease-relevant tissue to characterize important pathomechanisms.^52^ Increasing understanding of the regulatory features in intergenic DNA^53^, ^54^ provides insight into possible effector genes, either locally or distant from variants identified by GWAS. Skin as an organ that can be cultured *in vitro* offers an opportunity to investigate genetic mechanisms using primary cells, quantification of barrier function, and detailed molecular analyses. Cells extracted from normal human skin have been used in our model to investigate the effect of a single candidate gene without the multiple genetic and epigenetic effects that would be coinherited in cells harvested from a patient with AD.^2^ Use of primary cells more closely represents skin physiology than an immortalized cell line, and replication in multiple donors is used to control for interindividual variation.

Our findings from genomic data and *in vitro* and *ex vivo* analyses converge to provide an understanding of the role of EMSY in skin barrier function. The epigenetic regulatory mechanisms indicated by methylation and chromosomal confirmation (Fig 1) provide a possible mechanism by which the intergenic SNPs associated with AD can affect EMSY expression. However, additional epigenetic mechanisms,^55^ including other forms of histone modification, micro-RNAs, and long noncoding RNAs, might also play roles in *EMSY* transcriptional control. The greater nuclear localization of EMSY in lesional skin of patients with AD indicates increased activity of this transcriptional repressor. This is in keeping with our findings in primary cell cultures, where *EMSY* overexpression leads to a reduction in levels of multiple proteins that have previously been shown to be biomarkers for AD.^56^, ^57^, ^58^ Improvements in analytic capacity have increased understanding of the importance of lipid composition in epidermal physiology and pathophysiology, including atopic disease,^59^ and the observed increase in relevant lipid species emphasizes the role of EMSY in controlling multiple aspects of skin barrier function.

It is interesting to note that *EMSY* knockdown increases filaggrin expression, whereas *EMSY* overexpression leads to a marked reduction in profilaggrin levels. This might in part explain the observation that the genetic risk variants in chromosome 11q13.5 and *FLG* show a multiplicative effect in population analysis.^60^ Chromosome 11q13.5 and the *FLG* locus also both show their strongest associations within a subgroup of early-onset and persistent AD in childhood,^61^ which is in keeping with their combined effect leading to a more severe phenotype.

*EMSY* loss-of-function mutations are rare: they are detected in 1/60,000 unrelated subjects in the Exome Aggregation Consortium,^62^ the minor allele frequency is approximately 0.001 in the Exome Variant Server,^63^ and there are no homozygous loss-of-function genotypes identified in gnomAD.^64^ This is consistent with our finding that even a modest reduction in expression (mean approximately 33% reduction in mRNA or protein in our experimental model) results in a marked phenotypic change in organotypic cultures.

Together, these observations indicate that loss-of-function mutations are likely to have a deleterious effect, and more subtle modulation of *EMSY* expression is required for optimal skin barrier function. Therefore it is tempting to speculate that the control of *FLG* expression and skin barrier function through EMSY might be a more sensitive mechanism to exploit than targeting filaggrin directly. It might also be a more tractable target because the therapeutic effect would be achieved by knockdown of EMSY rather than attempting to increase expression of filaggrin.

Loci reaching statistical significance in genome-wide analyses might reflect the combination of more than 1 functional association, and multieffect loci have been observed in patients with AD, as well as other complex traits.^12^, ^65^ Our finding of a role for *EMSY* in keratinocytes does not preclude an additional effect of *EMSY* through expression in T cells,^66^ which are known to play a key role in AD pathogenesis.^2^ Intriguingly, immunohistochemistry also reveals EMSY staining within the nuclei of cells in the dermis (Fig 6 and see Fig E10). This might represent an inflammatory infiltrate, including T cells, and warrants further investigation. Similarly, the genomic promoter-capture Hi-C analysis (see Fig E1) provides more support for a possible promoter-promoter interaction between the GWAS locus and *LRRC32* than *EMSY*, suggesting that an effect through *LRRC32* expression in keratinocytes or T cells might also play a role in the pathogenesis of AD. Further work, including high-resolution 3C,^67^ could be used to finely map these important regulatory interactions.

Expression quantitative trait locus analysis is an approach that has been used to investigate genes responsible for risk mechanisms attributable to intergenic loci.^68^ However, expression quantitative trait locus analysis relies on the genetic variants having a quantitative effect on mRNA abundance of sufficient magnitude to be detected with a suitably stringent level of statistical significance.^69^ Other mechanisms can affect disease risk without substantially altering the total amount of mRNA, such as protein localization at a functional site, as appears to be the case for *EMSY*. Furthermore, *EMSY* has at least 7 isoforms produced by alternative splicing, as well as 14 annotated phosphorylation or glycosylation sites (https://www.uniprot.org/uniprot/Q7Z589); it is likely that these mechanisms also contribute to functional regulation in different cell and tissue types.

Analysis of a single cell type, the keratinocyte, at different stages of terminal differentiation represents a reductionist view of the epidermis, but the skin organotypic model has proved to be a valuable model for investigation of molecular mechanisms controlling differentiation in this multilayered tissue.^40^, ^70^ A limitation to the model is that the various dermal appendages (eg, hair follicles, sweat glands, and sebaceous glands) are absent, as are the dermal blood vessels and neurons, although these might play a role in skin inflammation.^71^, ^72^ Immune cells of the hematopoietic lineage are also missing from this model, but there is increasing recognition of the role of keratinocytes in immune signaling,^73^, ^74^ and the organotypic culture displays innate immunologic mechanisms.

The work presented here has identified EMSY as a therapeutic target: knockdown *in vivo* is predicted to improve skin barrier function and protect against AD. However, considerable further work is needed, including high-throughput screening to identify molecules capable of reducing the abundance of nuclear EMSY, followed by preclinical testing, before these findings can be assessed in clinical trials. The advent of biological therapy to target the immune component of AD has transformed care for patients with moderate-to-severe disease,^75^ but therapies designed to improve skin barrier function are also required as an alternative or complementary approach for this complex and therapeutically challenging disorder.

**Key messages**

• Genetic risk loci offer the opportunity for insight into the cause of complex disease, but the mechanisms require detailed molecular investigation.
• The AD-associated locus on chromosome 11q13.5 lies between 2 candidate genes: *EMSY* and *LRRC32.*
• Our genetic, proteomic, and immunohistologic analyses have together demonstrated a role for *EMSY* expression in the control of skin barrier formation and function, which are of importance in patients with AD.
• Therefore EMSY represents a future therapeutic target in atopic disease.

## Supplementary data

Online Repository text

Figs E1-E7

Fig E8

Figs E9 and E10
