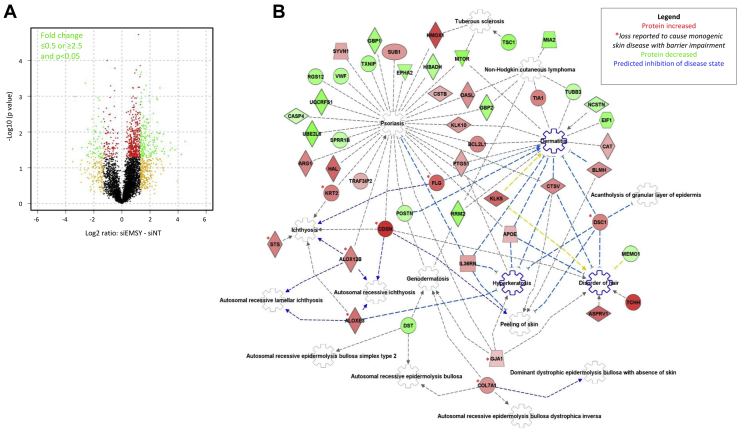# Corrigenda

**DOI:** 10.1016/j.jaci.2019.11.027

**Published:** 2020-02

With regard to the article in the August 2019 issue entitled “EMSY expression affects multiple components of skin barrier with relevance to atopic dermatitis” (J Allergy Clin Immunol 2019; 144:470-81), the authors report that they have identified an error in the method that was used for plotting data in Fig 5, *A*. The correct method is as follows:

“A volcano plot was generated in R/ggplot2 using human proteins detected in all 4 of the replicates. The log2 ratio (log2 EMSY knockdown – log2 NT control) for each donor was calculated and averaged; *P*-values from paired t-test were derived from log10 transformed data and are unadjusted.” The corrected Fig 5, *A* is shown below; correction of this figure does not impact the results, conclusions, or interpretation of the paper.

**Figure:**