# Supplementary material for: EMSY expression affects multiple components of the skin barrier with relevance to atopic dermatitis
Source: J Allergy Clin Immunol. 2019 Aug;144(2):470–81. doi: 10.1016/j.jaci.2019.05.024 (PMC6683598; doi:10.1016/j.jaci.2019.05.024)
Supplement: Online Repository text [file mmc1.doc]

**SUPPLEMENTARY METHODS**

**Promoter-capture Hi-C data analysis**

Fastq files from Rubin *et al*.^1^ were processed with the HiCUP pipeline version 0.6.1 (https://www.bioinformatics.babraham.ac.uk/projects/hicup/) which truncated, mapped, filtered and de-duplicated reads. Reference genome GRCh37 was used for mapping. Loci containing five AD-associated SNVs from the GWAS catalogue were investigated (rs11236809, rs7927894, rs2155219, rs2212434 and rs7130588) and genes within 250kb. Six baits within regions of 100kb around each SNP and genes of interest were identified. Bait-region interaction plots were generated for each of the baits using BioConductor package CHiCAGO^2^ version 1.10.1

**RT-qPCR**

RNA was extracted from organotypic epidermis using the Direct-zol RNA kit (Zymo Research, Irvine, California, USA) following homogenisation in RNA Bee (Amsbio, Abingdon,Oxfordhire, UK) using the TissueLyser LT (Qiagen, Manchester, UK) 5 mins at 50 Hz. cDNA was prepared from 1μg total RNA using random priming (2.5µM final) (Integrated DNA Technologies, Coralville, Iowa, USA) in combination with Moloney murine leukemia virus enzyme (MMLV) (100 units) and buffer systems (Life Technologies, Carlsbad, California, USA). qPCR reactions were performed using exon spanning probe-based assays (**Table E1,** below) in combination with TaqMan gene expression Mastermix (Life Technologies, Carlsbad, California, USA). Reactions were prepared and run using the Qiagility robot in combination with the Rotorgene Q (Qiagen, Manchester, UK). *EF1A* was used as a reference gene and fold changes were derived via the 2(-Delta Delta C(T)) method.

**Immunoblotting**

Organotypic epidermis was homogenized in RIPA buffer (Cell Signalling Technologies, London, UK) using the TissueLyser LT (Qiagen, Manchester, UK) 5 mins at 50 Hz. Protein lysates were normalized by Pierce BCA assay (Thermo Fisher, Waltham, Massachussets, USA) and resolved under reducing conditions using the NuPage® gel electrophoresis system (Life Technologies, Carlsbad, California, USA). Primary antibodies were prepared in 5% bovine serum albumin (BSA) and incubated overnight at 4^o^C with agitation (**Table E2,** below). Immunoblots were developed using peroxidase conjugated secondary antibodies (DAKO, Glostrup, Denmark, 1:5000) in combination with Chemiluminescent HPR substrate (Immobilon, Millipore, Billerica, Massachussets, USA) onto photographic film. Densitometry was performed using ImageJ.

**Image analysis for measurement of epidermal thickness**

Histological sections of 10 replicate skin organotypic experiments were measured using a standardized technique to minimize bias, as follows: Three points were measured on each slide, at the right side, middle and left of each sample, across viable cell layers and stratum corneum; measurements were generated using ImageJ and the mean of the three measurements calculated.

**Immunohistochemistry and immunofluorescence**

Formalin-fixed, paraffin embedded normal skin and AD samples were processed in unison for immunohistochemistry. Staining was performed using the automated EnVision+ system in combination with DAB detection and haematoxylin counterstain (DAKO, Tayside Tissue Bank service). Slides were imaged using the automated Aperio ImageScope slide scanner allowing deep zoom magnification (Leica Biosystems, Wetzlar, Germany Biosystems). Formalin-fixed, paraffin embedded full thickness organotypics were processed by immunofluorescence under standard conditions using an Alexa Fluor 568 conjugated secondary antibody and DAPI counterstain (Life Technologies, Carlsbad, California, USA). Negative control sections were stained in the absence of a primary antibody. Slides were imaged by confocal Zeiss LSM710 microscope (Carl Zeiss Microscopy GmbH, Jena, Germany). Antibodies are detailed in **Table E3** (below).

**Transmission electron microscopy**

Organotypic skin samples were fixed in 4% paraformaldehyde, 2.5% gluteraldehyde in 0.1M sodium cacodylate buffer (pH 7.2) for one hour then cut into small pieces, washed in buffer and post-fixed in 1% osmium tetroxide in cacodylate buffer for one hour. The pieces were dehydrated through alcohol series, into propylene oxide and embedded in Durcupan resin. Ultrathin sections were stained with 3% aqueous uranyl acetate and Reynold’s lead citrate and examined on a JEOL 1200EX electron microscope. Images were collected on a SIS Megaview III camera.

**Mass spectrometry proteomic analysis**

Frozen organotypic epidermal samples were ground on dry ice, solubilized in 200μl of Cellular and Organelle Membrane Solubilizing Reagent (Sigma-Aldrich, Gillingham, Dorset, UK), 7.0 M urea, 2.0 M thiourea, 40 mM Trizma® base (Sigma Aldrich, Gillingham, Dorset, UK) and 1.0% C7BzO, pH 10.4 buffer with protease inhibitors. The lysate was acetone precipitated for 1 hour at -20^o^C and spun for 20 mins at 2^o^C at 15,000 rpm. The pellet was re-suspended in 200μl of 50mM ammonium bicarbonate. 100μl was taken for in-solution digest. 22μl of dithiothreitol was added and samples heated to 50^o^C for 15mins. 24μl of iodoacetamide was added and the samples incubated at 22^o^C for 30 mins. 1μl of Rapigest was added, followed by 2.5μl of trypsin, for a 12-hour digest. A further 1μl trypsin was added for an additional 4-hour digest. The peptide samples were fractionated into 24 fractions with high pH Reversed Phase C18 chromatography, run on a Q Exactive Classic or Plus for 160 mins and the top 15 ions selected for sequencing. MS resolution was 70,000 and MS/MS resolution 17,500. Data were processed using MaxQuant (v1.6.0.13) and Human UniProt Database (Dec 2017) with a protein and peptide false discovery rate of 0.01.

Proteins identified as contaminants (trypsin and other lab originating proteins) and contained within the MaxQuant database (http://www.maxquant.org/maxquant/) were removed. To account for the possibility of keratin as a contaminant, we analysed the blank runs for keratin peptide intensity and subtracted this from the measured keratin intensities. A reversed database (i.e. scrambled protein sequences) was used to identify false positives and these were also removed. Total intensities of the samples were normalized to total protein abundance, with adjustment to the lowest total protein yield. All samples were run in quadruplicate to test reproducibility; the Pearson correlations were 0.86-0.92 (**Fig. E6**).

**Mass spectrometry lipidomic analysis**

Skin samples were sonicated in phosphate buffered saline (pH7.4) and then extracted according to the method of Folch *et al.*^3^. Ceramide 17:0 (Avanti Polar Lipids, Alabaster, AL, USA) was included in the experimental system as an internal standard. The skin lipids were analysed by liquid chromatography-mass spectrometry (LC-MS) on a Thermo Exactive Orbitrap mass spectrometer (Thermo Scientific, Hemel Hempsted, UK) coupled to a Thermo Accela 1250 ultra high pressure liquid chromatography (UHPLC) system. Samples were injected on to C18 column (Thermo Hypersil Gold, 2.1 mm x 100 mm, 1.9 μm) and separated using a water/acetonitrile/isopropanol gradient^4^. Ion signals corresponding to individual ceramide molecular species were extracted from raw LC-MS data sets. The concentration of each ceramide was expressed as pmol/mg after normalisation to mg of wet weight tissue. Chain lengths reported in our data correspond to ceramides reported in AD skin *in vivo* as follows: Cer22 relates to NS CER 40 carbons in work by van Smeden *et al* ^5^ and esterified omega-hydroxy ceramide d18:1/50:2 species are equivalent of acyl-CER with 68 carbons, each of which show reduced abundance in AD skin *in vivo*^5^.

**Lipid data analysis**

Ceramide and esterified omega-hydroxy-ceramide data were analysed separately and matched samples were compared using replicate experimental data without pooling. The ratio of lipid concentration was computed, to compare *EMSY* siRNA-treated organotypic with donor-matched control sample; results were log transformed using natural log (log_e_). To investigate a trend in differential expression dependent on lipid length, a linear model was fitted and plotted with 95% confidence intervals; p-values were computed for the term in the linear model for chain length, where p<0.05 indicates a significant difference in lipid length between *EMSY* siRNA-treated samples and controls. Analyses were performed with R-style formula “<logged-expression-ratio> ~ <lipid-length> + <sample>” and the glm function in R (version 3.5.1); data and the linear models were plotted with the R package ggplot2 (version 3.1.0).

**Clinical samples used for immunohistochemistry**

Control skin was obtained from adult patients, with written informed consent, as surplus tissue generated from routine surgical procedures. Male and female patients, with clinically normal skin were used. Individuals having undergone systemic chemotherapy were excluded.

AD skin samples were obtained as surplus to clinical care and used with written informed consent. Demographic and phenotypic features of these patients are shown in **Table E4** (below). Samples were identified from the pathology database by searching for the histological diagnosis of eczema or dermatitis, with spongiosis; samples with any features of other forms of dermatitis (eg stasis changes, marked eosinophilic infiltration, photodamage or signs of external trauma) were not selected for the study. There was no apparent correlation between skin site, gender, age or AD severity and EMSY immunostaining.

| **GENE NAME** | **ASSAY ID/SEQUENCE** | **CONCENTRATION** |
| --- | --- | --- |
| ***FLG*** | HS.PT.58.24292320 (FAM/ZEN/IBFQ) | PRIMERS: 500 NM |
|  | (Integrated DNA Technologies) | PROBE: 250 NM |
| ***EMSY*** | Hs.PT.58.28326190 (FAM/ZEN/IBFQ) | PRIMERS: 500 NM |
|  | (Integrated DNA Technologies) | PROBE: 250 NM |
| ***EF1A*** | Hs.PT.58.24345862 (FAM/ZEN/IBFQ) | PRIMERS: 500 NM |
|  | (Integrated DNA Technologies) | PROBE: 250 NM |
| ***CDSN*** | F: ATGATGGCACTGCTGCTG | PRIMERS: 400 NM |
|  | R: AAGGTGCCAATGCTCTTAGC | PROBE: 100 NM |
|  | P: UPL#51 |  |
| ***DSC1*** | F: GGGGCAGGGAGATACTGG | PRIMERS: 400 NM |
|  | R: CAGAGTGTGTCCTCTAATGGATTC | PROBE: 100 NM |
|  | P: UPL#53 |  |
| ***FLG2*** | F: CTGACTATGGCCTGCAACAA | PRIMERS: 400 NM |
|  | R: CTTTGACCCTGAAGCTTTGC | PROBE: 100 NM |
|  | P: UPL#73 |  |
| ***K2*** | F: GCCTCCTTCATTGACAAGGT | PRIMERS: 400 NM |
|  | R: GCTGTCGATATACCCCTGGA | PROBE: 100 NM |
|  | P: UPL#1 |  |
| ***HAL*** | F: GAACTGAACAGCGCAACAGA | PRIMERS: 400 NM |
|  | R: TTTGGCTGGGTATTCACCAT | PROBE: 100 NM |
|  | P: UPL#65 |  |
| ***BLMH*** | F: AGGGAACATGTCAAGCCACT | PRIMERS: 400 NM |
|  | R: TTCCACTGTGTAAAGTTTGTTGTACTT | PROBE: 100 NM |
|  | P: UPL#57 |  |
| ***ASPRV1*** | F: TCTGTGGTCCACCCAAACTT | PRIMERS: 400 NM |
|  | R: GCTGCAGGGTGTCCAGAT | PROBE: 100 NM |
|  | P: UPL#52 |  |
| ***ALOXE3*** | F: CAAGAACCCAAGGACCAGAG | PRIMERS: 400 NM |
|  | R: TCCTGGATGTCCCTTGAGAT | PROBE: 100 NM |
|  | P: UPL#82 |  |
| ***ALOX12B*** | F: GCTACGAGACCCTGGCACT | PRIMERS: 400 NM |
|  | R: AGAAGTCCTGCTTGGCTCTG | PROBE: 100 NM |
|  | P: UPL#1 |  |
| ***STS*** | F: CATGGACATATTTCCTACAGTAGCC | PRIMERS: 400 NM |
|  | R: CACGTCCATCAATGATCCTGT | PROBE: 100 NM |
|  | P: UPL#19 |  |
| ***APOE*** | F: CAGGCAGGAAGATGAAGGTT | PRIMERS: 400 NM |
|  | R: CTGTCTCCACCGCTTGCT | PROBE: 100 NM |
|  | P: UPL#72 |  |
| ***COL7A1*** | F: GCTGGTGCTGCCTTTCTCT | PRIMERS: 400 NM |
|  | R: TCCAGGCCGAACTCTGTC | PROBE: 100 NM |
|  | P: UPL#71 |  |
| ***MTOR*** | F: TTTAGCGGTCATGTCAATGG | PRIMERS: 400 NM |
|  | R: CATCAGGTTGGATGGGTGT | PROBE: 100 NM |
|  | P: UPL#14 |  |
| ***IL36RN*** | F: GCAGGGAAGGTCATTAAAGGT | PRIMERS: 400 NM |
|  | R: CCTGGACACCCAGGATGA | PROBE: 100 NM |
|  | P: UPL#42 |  |
| ***CDK1*** | F:TGGATCTGAAGAAATACTTGGATTCTA | PRIMERS: 400 NM |
|  | R:TCTGGAGATCTGTACCAGAGTGTT | PROBE: 100 NM |
|  | P: UPL#79 |  |
| ***RNASE7*** | F:GAAGACCAAGCGCAAAGC | PRIMERS: 400 NM |
|  | R:AGCAGAAGGGGGCAGAAT | PROBE: 100 NM |
|  | P: UPL#63 |  |
| ***GJA1*** | F:GCCTGAACTTGCCTTTTCAT | PRIMERS: 400 NM |
|  | R: CTCCAGTCACCCATGTTGC | PROBE: 100 NM |
|  | P: UPL#88 |  |

**Table E1. qPCR assay details**
F: forward primer, R: reverse primer, P: probe, UPL: Universal Probe Library and corresponding probe number (#) (Sigma Aldrich).

| **PROTEIN NAME** | **MANUFACTURER / CLONE** | **1° ANTIBODY DILUTION** |
| --- | --- | --- |
| **FILAGGRIN** | **Santa Cruz** | **1/250** |
|  | **sc66192** |  |
| **EMSY** | **Bethyl Laboratories** | **1/2000** |
|  | **A300-253A** |  |
| **KERATIN, TYPE II CYTOSKELETAL 2 EPIDERMAL** | **Abcam** | **1/1000** |
|  | **ab170106** |  |
| **HISTADINE**  **AMMONIA-LYASE** | **Thermo Fisher** | **1/800** |
|  | **PA1-518** |  |
| **SERINE/THREONINE-PROTEIN KINASE MTOR** | **Thermo Fisher** | **1/500** |
|  | **PA1-518** |  |
| **ARACHIDONATE**  **12-LIPOXYGENASE** | **Thermo Fisher** | **1/1000** |
|  | **PS5-23608** |  |
| **CORNEODESMOSIN** | **Thermo Fisher** | **1/200** |
|  | **PA5-47768** |  |
| **DESMOCOLLIN-1** | **Novus** | **1/1000** |
|  | **NBP-1-88099** |  |
| **FILAGGRIN-2** | **Abcam** | **1/500** |
|  | **ab122011** |  |
| **STERYL-SULFATASE** | **Abcam** | **1/1000** |
|  | **ab62219** |  |
| **HYDROPEROXIDE ISOMERASE ALOXE** | **Thermo Fisher** | **1/3000** |
|  | **PA5-2183** |  |
| **GAP JUNCTION**  **ALPHA-1 PROTEIN** | **Thermo Fisher** | **1/500** |
|  | **71-0700** |  |
| **INTERLEUKIN-36**  **RECEPTOR ANTAGONIST** | **ATLAS** | **1/200** |
|  | **HPA034542** |  |
| **GYLYCERALDEHYDE-**  **3-PHOSPHATE DEHYDROGENASE** | **Cell signalling** | **1/10,000** |
|  | **97166** |  |

**Table E2. Immunoblotting antibody details**

| **PROTEIN NAME** | **MANUFACTURER / CLONE** | **1° ANTIBODY DILUTION** | **HIER** |
| --- | --- | --- | --- |
| **FILAGGRIN** | **Santa Cruz** | **1/1000** | **TRIS/EDTA** |
|  | **sc66192** |  |  |
| **EMSY** | **Bethyl Laboratories** | **1/250** | **Tris/EDTA** |
|  | **A300-253A** |  |  |
| **KERATIN, TYPE II CYTOSKELETAL 2 EPIDERMAL** | **Thermo Fisher** | **1/500** | **CITRATE** |
|  | **PA5-21456** |  |  |
| **HISTADINE**  **AMMONIA-LYASE** | **ATLAS** | **1/500** | **CITRATE** |
|  | **HPA038547** |  |  |
| **SERINE/THREONINE-PROTEIN KINASE MTOR** | **Thermo Fisher** | **1/200** | **CITRATE** |
|  | **PA1-518** |  |  |
| **ARACHIDONATE 12-LIPOXYGENASE** | **Thermo Fisher** | **1/200** | **CITRATE** |
|  | **PS5-23608** |  |  |
| **CORNEODESMOSIN** | **Thermo Fisher** | **1/200** | **CITRATE** |
|  | **PA5-47768** |  |  |
| **DESMOCOLLIN-1** | **Novus** | **1/200** | **CITRATE** |
|  | **NBP-1-88099** |  |  |

**Table E3. Immunohistochemistry and immunofluorescence antibody details**
HIER: heat induced epitope retrieval method

| **Age (years)** | **Sex** | **Site of biopsy** | **Clinical features** | **Histological diagnosis** |
| --- | --- | --- | --- | --- |
| 28 | Female | right abdominal skin | itchy lesion | subacute dermatitis |
| 66 | Female | areolar | severe eczema | dermatitis, eczematous |
| 50 | Female | areolar | eczema | very occasional eosinophils |
| 23 | Female | areolar | eczema | dermatitis, eczematous |
| 29 | Female | areolar | mild eczema | dermatitis, eczematous |
| 60 | Female | skin overlying left hip | atopic eczema and widespread flare | spongiotic dermatitis |
| 49 | Male | right neck | atopic background | area of chronic dermatitis |
| 49 | Female | right cheek | itchy lesion | mild spongiosis, chronic dermatitis |
| 64 | Female | areolar | moderately severe eczema | dermatitis, eczematous |
| 19 | Female | right inner upper arm | patchy dry rash | mild chronic dermatitis |
| 62 | Male | mid-back | itchy | mild spongiosis, mild chronic dermatitis |
| 48 | Female | right medial wrist | atopic eczema | chronic spongiotic dermatitis |
| 55 | Male | left forearm | erythroderma | mild spongiosis, subacute dermatitis |
| 24 | Female | right flank | atopic dermatitis flare | chronic spongiotic dermatitis |

**Table E4. Demographic and phenotypic features of patients from whom skin biopsy samples were obtained.**

**References**

1. Rubin AJ, Barajas BC, Furlan-Magaril M, Lopez-Pajares V, Mumbach MR, Howard I, et al. Lineage-specific dynamic and pre-established enhancer-promoter contacts cooperate in terminal differentiation. Nat Genet 2017; 49:1522-8.

2. Cairns J, Freire-Pritchett P, Wingett SW, Varnai C, Dimond A, Plagnol V, et al. CHiCAGO: robust detection of DNA looping interactions in Capture Hi-C data. Genome Biol 2016; 17:127.

3. Folch J, Lees M, Sloane Stanley GH. A simple method for the isolation and purification of total lipides from animal tissues. J Biol Chem 1957; 226:497-509.

4. McIlroy GD, Tammireddy SR, Maskrey BH, Grant L, Doherty MK, Watson DG, et al. Fenretinide mediated retinoic acid receptor signalling and inhibition of ceramide biosynthesis regulates adipogenesis, lipid accumulation, mitochondrial function and nutrient stress signalling in adipocytes and adipose tissue. Biochem Pharmacol 2016; 100:86-97.

5. van Smeden J, Janssens M, Kaye EC, Caspers PJ, Lavrijsen AP, Vreeken RJ, et al. The importance of free fatty acid chain length for the skin barrier function in atopic eczema patients. Exp Dermatol 2014; 23:45-52.

**Supplementary Figure Legends**

**Figure E1. Chromosomal conformation data analysis**

**a**. Data from Rao *et al.* (2014) generated using normal human keratinocytes, displayed in the Hi-C data browser from Yue lab, Penn State, USA

http://promoter.bx.psu.edu/hi-c/view.php?method=Hi-C&species=human&assembly=hg19&source=inside&tissue=NHEK&type=Liebermanraw&

resolution=5&c_url=&transfer=&gene=&chr=chr11&start=75553333.333333&end=76871666.666667&sessionID=&browser=none

**b.** Data from Rubin *et al.* (2017) generated using normal human keratinocytes in duplicate experiments. Results are shown for day 0; cells differentiated to days 3 and 6 gave very similar results. Coloured rectangles indicate position of the genes and blue lines show the SNP positions.

The vertical red line is the location of the bait. Plots shows read count added across the two replicate experiments. There is some evidence of an interaction between the SNP region and *LRRC32,* but an interaction with *EMSY* cannot be excluded.

**Figure E2. Direct (single molecule) RNA sequencing of atopic skin compared to non-atopic control skin**

Direct RNA sequencing analysis (48) of skin biopsies from the clinically uninflamed skin of paediatric AD patients (n=26) and site-matched non-atopic control skin (n=10); box plots show median, first and third quartiles, whiskers extend to most extreme data point <1.5 x interquartile range; p>0.05

Details of the donor phenotypes and demographic details are reported in Cole *et al.* (2014).

**Figure E3. Comparison of effects of individual and pooled EMSY-siRNAs**

**a.** Bar chart showing mean EMSY mRNA; error bars show standard deviaEon; C4, non-targeting siRNA control, E5-E8, individual siRNAs, Epool, pooled treatment using E5-E8 in

equal amounts to create the same total concentration of siRNA; biological replicate experiments were performed, using primary keratinocytes from 5 different skin donors.

**b. Western blot**

**c.** Bar chart showing mean capacitance measured on the surface of skin organoid samples using a Corneometer (Courage-Khazaka Electronic, Köln); error bars show standard deviation; C4, non-targeting siRNA control, E5-E8, individual siRNAs, Epool, pooled treatment using E5-E8 in equal amounts to create the same total concentration of siRNA; biological replicate experiments were performed, using primary keratinocytes from 3 different skin donors.

**d. H&E stained secHons of skin organoid samples** Histological sections showing appearance of organoid samples untreated (mock) and treated with control (non-targeting siRNA), individual EMSY-siRNAs (E5, E6, E7, E8) and the pool of these four siRNAs in equal amounts to make the same concentration; similar findings were replicated in a total of three donors.

**Figure E4. Time course showing organoid development and measurements of barrier formation**

**a. H&E staining of skin organotypic cultures** Time after lifting culture to the air-liquid interface

**b. Measurement of barrier function** Water content of stratum corneum, measured using corneometer Transepidemal water loss (TEWL), measured using Aquaflux (Biox, London,

UK)

**c. Lucifer yellow dye penetration** Time after lifting culture to the air-liquid interface

50ul of 1mM dye placed on surface of organotypic skin contained within a metal ring

Incubated at 37oC for 4 hours

Image and image analysis performed with Zeiss LSM710 fluorescent microscope

[Note this figure is reproduced for comparison in Fig 3C.]

**Figure E5. EMSY over-expression in primary human keratinocytes leads to a reduction in markers of terminal differentiation and barrier function, consistent with changes previously reported to occur in AD.**

Data shown are from 3 biological replicates (primary kera8nocytes from 3 separate donors) in which EMSY mRNA was increased 10-fold, 22-fold and 47-fold compared to GFP-empty vector-treated kera8nocytes (**Figure 2J**).

**Figure E6. Mass spectrometry proteomic analysis quality control assessments.**

**a.** Bins on the x-axis contain the log2 intensity-based absolute quantification (iBAQ, sum of intensities of tryptic peptides for each protein divided by number of theoretically observable peptides); normalised counts of the proteins in each bin are shown on the y-axis; MS2, MS3, MS5 and MS6 are biological replicate experiments from different human donors; mock, untreated control organoid; C4, non-targe1ng control treated organoid; Emsy, EMSY si-RNA treated organoid.

**b.** x and y axes show iBAQ intensities of samples mapped against replicate analysis; biological replicates are highly correlated (Pearson correlation coefficient ≥0.86)

**Figure E7.**

**(a) Gene Ontology analysis of 154 proteins up-regulated in organoid skin with EMSY knock-down; results where FDR p<0.05**

**(b) Gene Ontology analysis of 130 proteins down-regulated in organoid skin with EMSY knock-down; results where FDR p<0.05**

Biological process and molecular func1on show no significant enrichments after FDR

**Figure E8. qPCR, Western blotting and immunofluorescence to test for validation of selected protein expression changes identified by mass spec analysis**

**Figure E9. Transmission electron microscopy images of organoid skin model with osmium staining, showing ultrastructure of stratum corneum**

EMSY knock-down produces a thicker stratum corneum (SC) with an increased number of component layers, consistent with the increase in corneodesmosin detected by mass spectrometry; black arrows indicate corneodesmosomes; representa-ve images from four

biological replicate experiments.

[Note: These upper two Images are reproduced here from Figure 2F, to allow comparison with replicate experiment.]

**Figure E10. Immunohistochemistry to investigate EMSY expression in skin**
