# Supplementary material for: EMSY expression affects multiple components of the skin barrier with relevance to atopic dermatitis
Source: J Allergy Clin Immunol. 2019 Aug;144(2):470–81. doi: 10.1016/j.jaci.2019.05.024 (PMC6683598; doi:10.1016/j.jaci.2019.05.024)
Supplement: Figs E1-E7 [file mmc2.pdf]

SUPPLEMENTARY FIGURES AND TABLES

EMSY expression affects multiple components of skin barrier with relevance to atopic dermatitis

Martina S Elias PhD,[1]* Sheila C Wright HNC,[1] Judit Remenyi PhD,[1] James C Abbott PhD[2], Susan E Bray PhD[3], Christian Cole PhD[2], Sharon Edwards MBChB[4], Marek Gierlinski PhD[2], Mateusz Glok[1], John A McGrath FRCP[5], William V Nicholson PhD[1], Lavinia Paternoster PhD[6], Alan R Prescott PhD[7], Sara Ten Have PhD[8], Phillip D Whitfield PhD[9], Angus I Lamond PhD[8] and Sara J Brown FRCPE[1,10]*

Fig E1. Chromosomal conformation data analysis

a Hi-C data browser (hg19) **b Promoter-capture Hi-C data analysis**

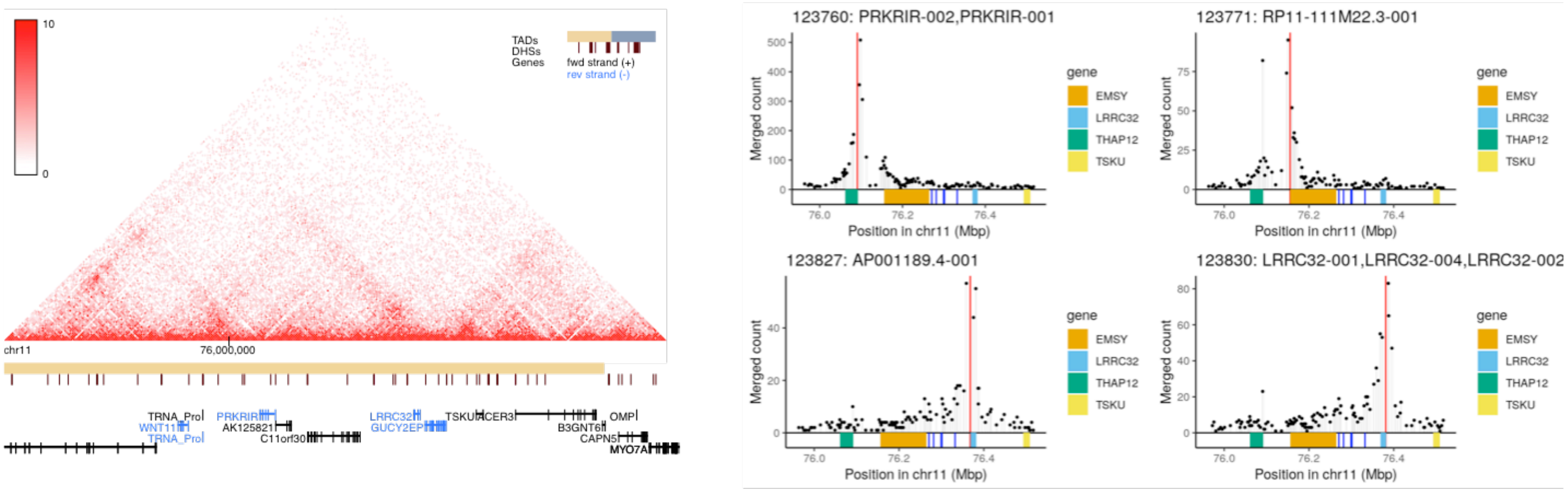

a. Data from Rao *et al.* (2014) generated using normal human keratinocytes, displayed in the Hi-C data browser from Yue lab, Penn State, USA http://promoter.bx.psu.edu/hi-c/view.php?method=Hi-C&species=human&assembly=hg19&source=inside&tissue=NHEK&type=Lieberman-raw&resolution=5&c_url=&transfer=&gene=&chr=chr11&start=75553333.333333&end=76871666.666667&sessionID=&browser=none

b. Data from Rubin *et al.* (2017) generated using normal human keratinocytes in duplicate experiments. Results are shown for day 0; cells differentiated to days 3 and 6 gave very similar results. Coloured rectangles indicate position of the genes and blue lines show the SNP positions. The vertical red line is the location of the bait. Plots shows read count added across the two replicate experiments. There is some evidence of an interaction between the SNP region and *LRRC32,* but an interaction with *EMSY* cannot be excluded.

Fig E2. Direct (single molecule) RNA sequencing of atopic skin compared to non-atopic control skin

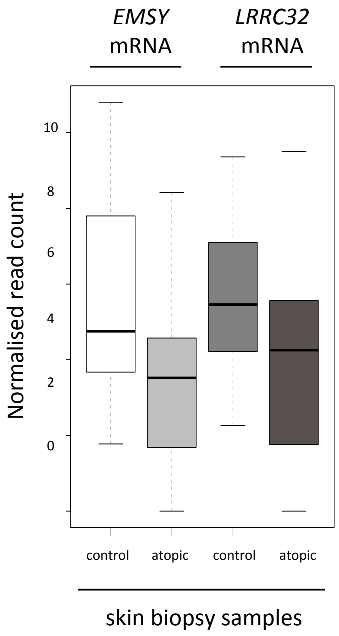

Direct RNA sequencing analysis (48) of skin biopsies from the clinically uninflamed skin of paediatric AD patients (n=26) and site-matched non-atopic control skin (n=10); box plots show median, first and third quartiles, whiskers extend to most extreme data point <1.5 x interquartile range; p>0.05

Details of the donor phenotypes and demographic details are reported in Cole *et al.* (2014).

Fig E3. Comparison of effects of individual and pooled *EMSY*-siRNAs

a

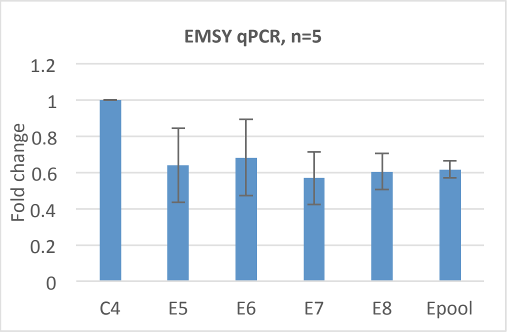

Bar chart showing mean *EMSY* mRNA; error bars show standard deviation; C4, non-targeting siRNA control, E5-E8, individual siRNAs, Epool, pooled treatment using E5-E8 in equal amounts to create the same total concentration of siRNA; biological replicate experiments were performed, using primary keratinocytes from 5 different skin donors.

b Western blot

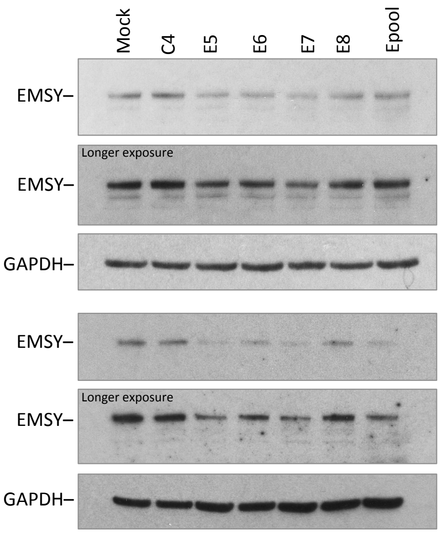

c

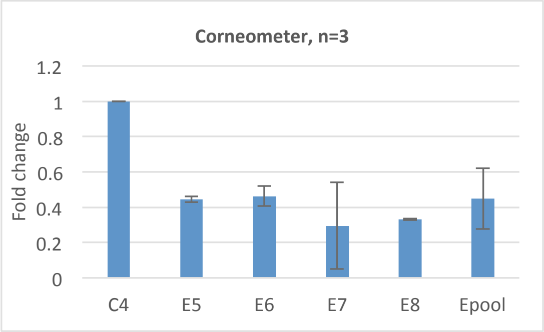

Bar chart showing mean capacitance measured on the surface of skin organoid samples using a Corneometer (Courage-Khazaka Electronic, Köln); error bars show standard deviation; C4, non-targeting siRNA control, E5-E8, individual siRNAs, Epool, pooled treatment using E5-E8 in equal amounts to create the same total concentration of siRNA; biological replicate experiments were performed, using primary keratinocytes from 3 different skin donors.

d H&E stained sections of skin organoid samples

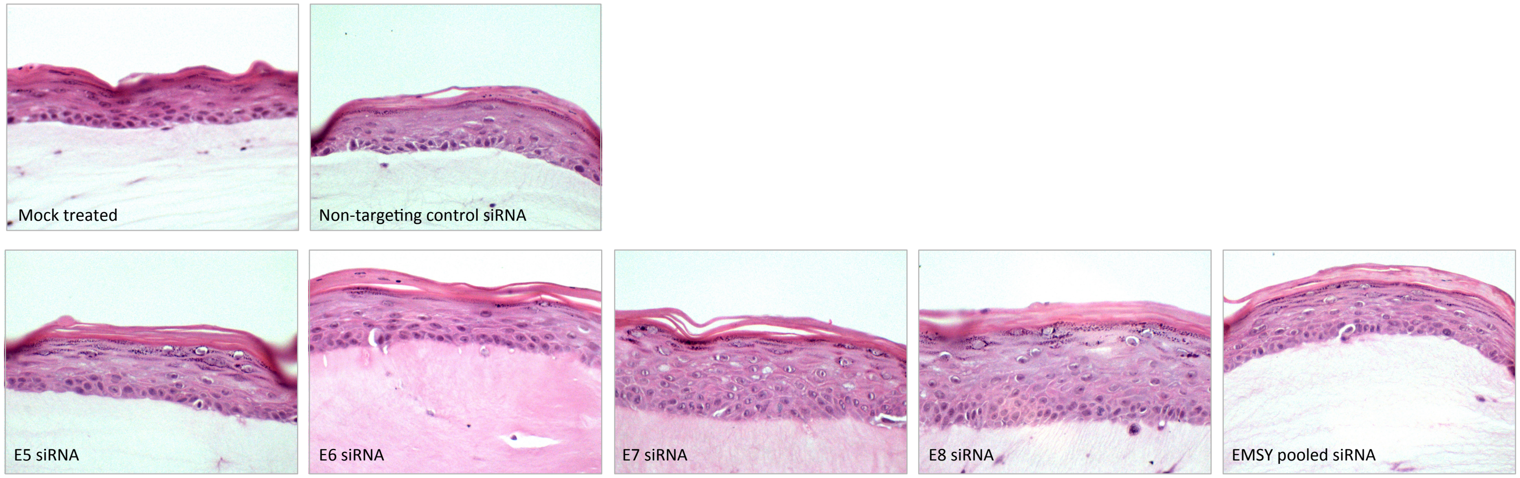

Histological sections showing appearance of organoid samples untreated (mock) and treated with control (non-targeting siRNA), individual EMSY-siRNAs (E5, E6, E7, E8) and the pool of these four siRNAs in equal amounts to make the same concentration; similar findings were replicated in a total of three donors.

Fig E4. Time course showing organoid development and measurements of barrier formation

a H&E staining of skin organotypic cultures

Time after lifting culture to the air-liquid interface

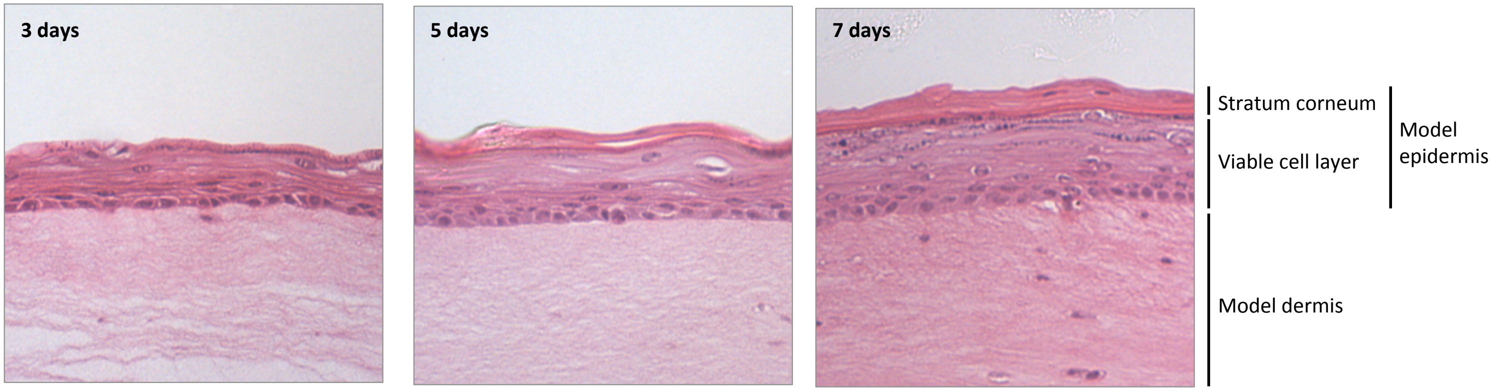

b Measurement of barrier function

Water content of stratum corneum, measured using corneometer

Transepidemal water loss (TEWL), measured using Aquaflux (Biox, London, UK)

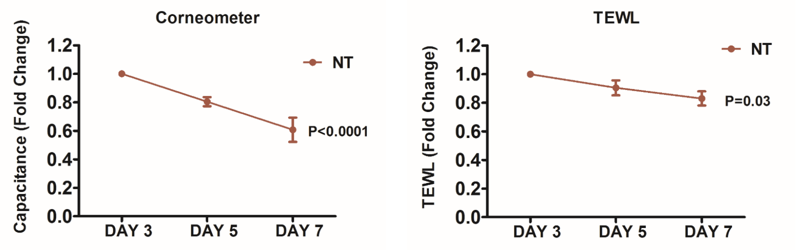

c Lucifer yellow dye penetration

Time after lifting culture to the air-liquid interface

50ul of 1mM dye placed on surface of organotypic skin contained within a metal ring

Incubated at 37ºC for 4 hours

Image and image analysis performed with Zeiss LSM710 fluorescent microscope

[Note this figure is reproduced for comparison in Fig 3C.]

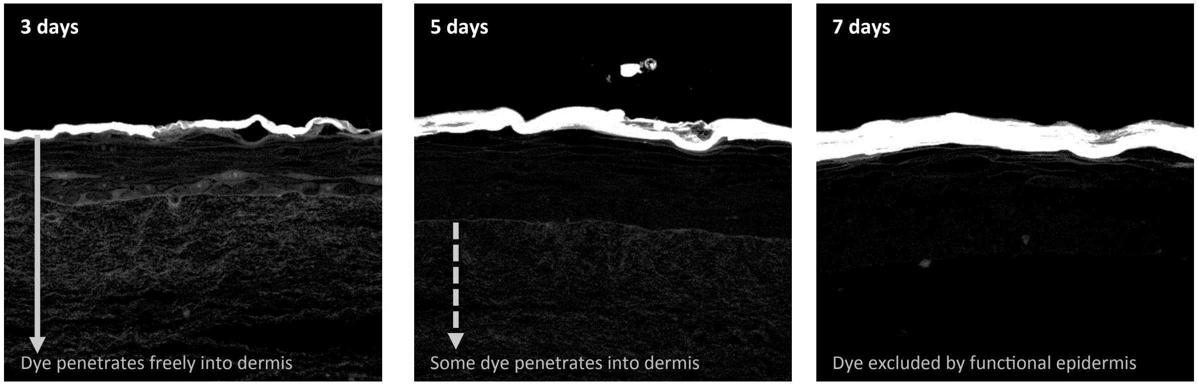

Fig E5. EMSY over-expression in primary human keratinocytes leads to a reduction in markers of terminal differentiation and barrier function, consistent with changes previously reported to occur in AD.

Data shown are from 3 biological replicates (primary keratinocytes from 3 separate donors) in which *EMSY* mRNA was increased 10-fold, 22-fold and 47-fold compared to GFP-empty vector-treated keratinocytes (**Figure 2J**).

a qPCR analysis

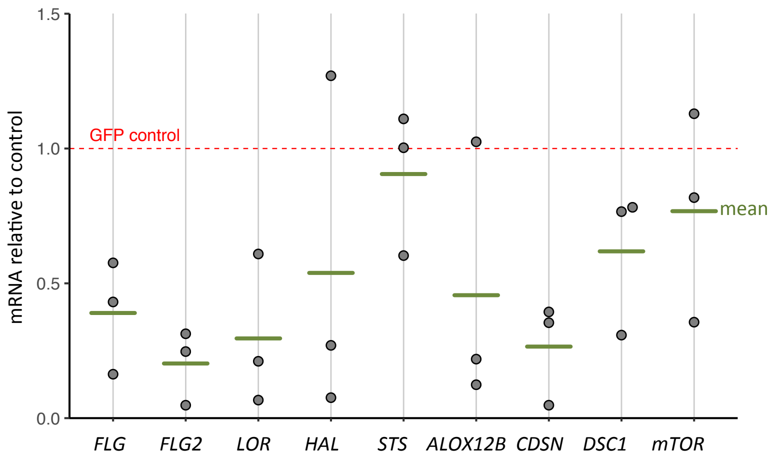

b Western blotting

Additional results from experiment shown in **Fig. 2l-2m** in which *EMSY* mRNA increased 47-fold

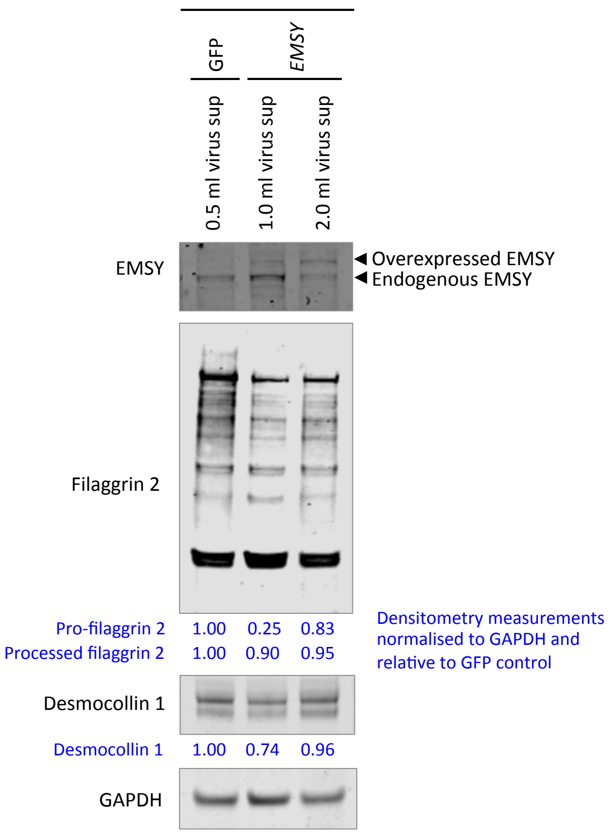

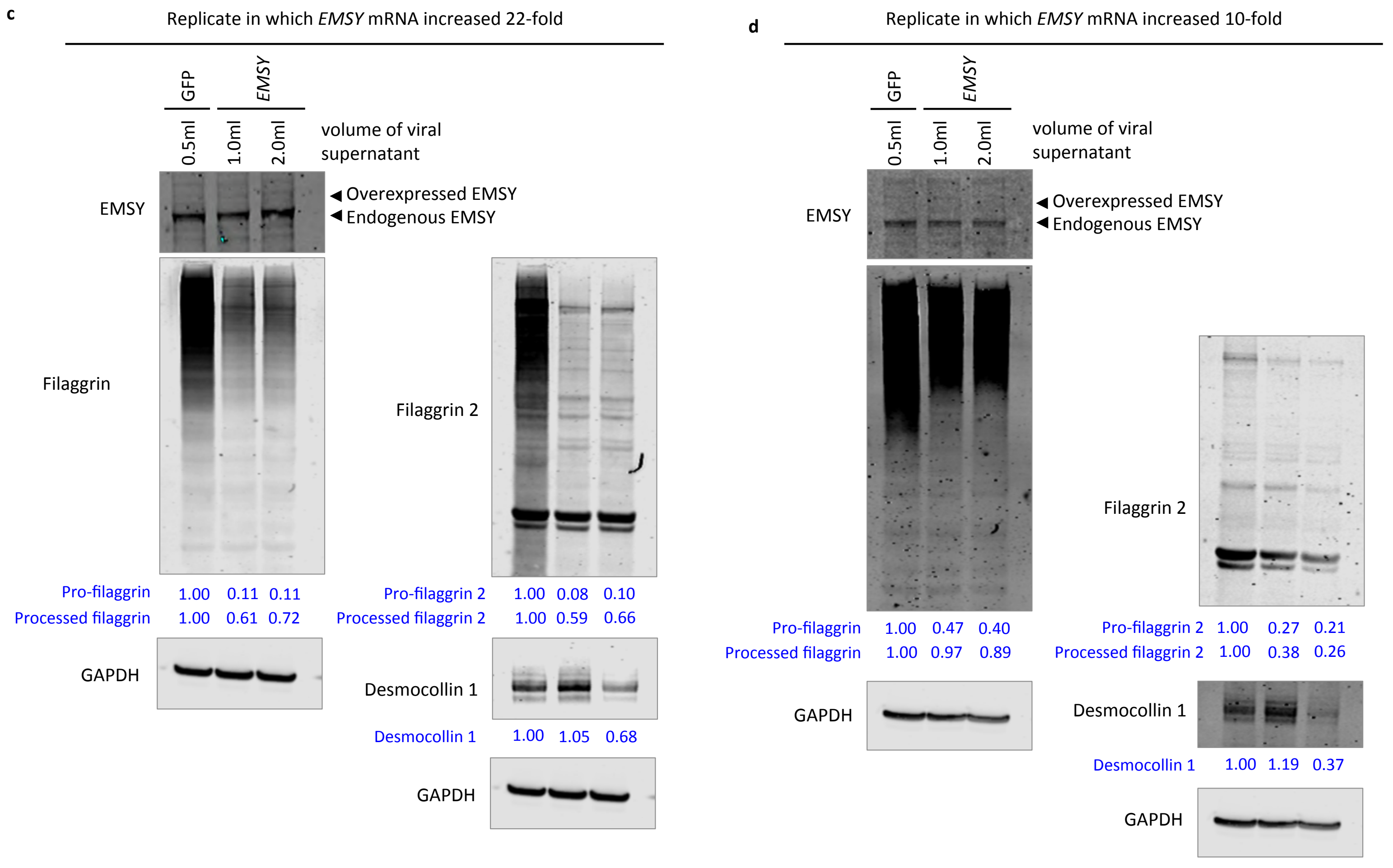

Densitometry measurements are normalised to GAPDH and relative to GFP control; ImageStudio

Fig E6. Mass spectrometry proteomic analysis quality control assessments.

a **Histograms showing distribution of proteins quantified in each experiment**

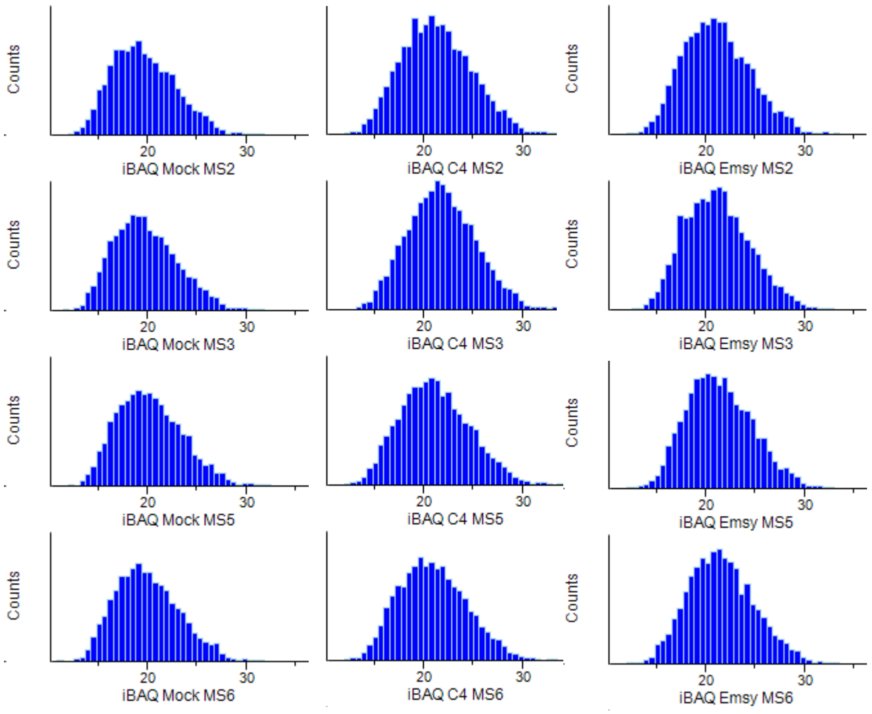

Bins on the x-axis contain the log2 intensity-based absolute quantification (iBAQ, sum of intensities of tryptic peptides for each protein divided by number of theoretically observable peptides); normalised counts of the proteins in each bin are shown on the y-axis; MS2, MS3, MS5 and MS6 are biological replicate experiments from different human donors; mock, untreated control organoid; C4, non-targeting control treated organoid; Emsy, EMSY si-RNA treated organoid.

b **Correlation of protein abundance from replicate analyses**

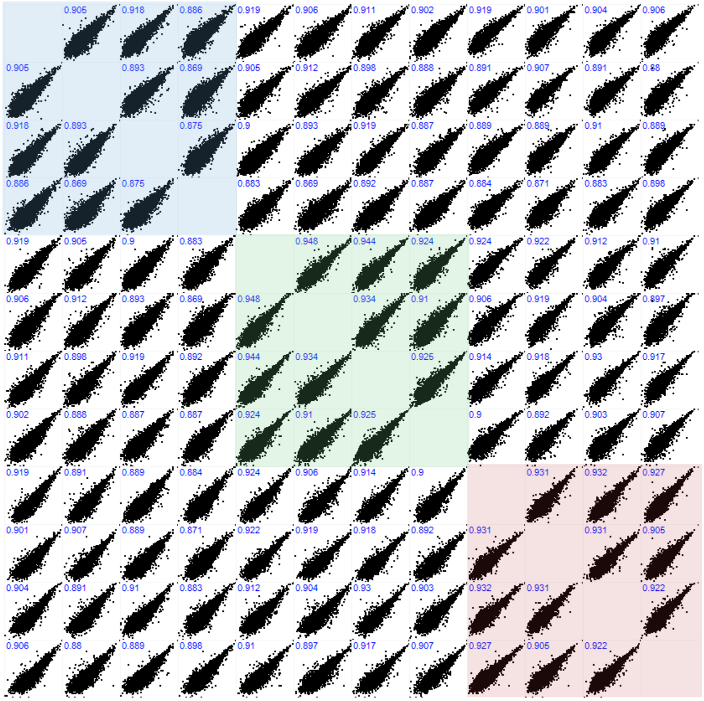

Non-targeting control

EMSY-siRNA treated

Mock-treated samples

x and y axes show iBAQ intensities of samples mapped against replicate analysis; biological replicates are highly correlated (Pearson correlation coefficient ≥0.86)

Fig E7.

(a) Gene Ontology analysis of 154 proteins up-regulated in organoid skin with EMSY knock-down; results where FDR p<0.05

Biological process

Analysis Type:	PANTHER Overrepresentation Test (Released 20181113)						
Annotation Version and Release Date:	GO Ontology database Released 2018-11-15						
Analyzed List:	upload_1 (Homo sapiens)						
Reference List:	Homo sapiens (all genes in database)						
Test Type:	FISHER						
Correction:	FDR						
GO biological process complete	Homo sapiens - REFLIST (21042)	upload_1 (161)	upload_1 (expected)	upload_1 (over/under)	upload_1 (fold Enrichment)	upload_1 (raw P-value)	upload_1 (FDR)
establishment of skin barrier (GO:0061436)	21	4	0.16	+	24.89	3.58E-05	3.14E-02
regulation of water loss via skin (GO:0033561)	23	4	0.18	+	22.73	4.90E-05	3.87E-02
cornification (GO:0070268)	112	7	0.86	+	8.17	3.51E-05	3.46E-02
cellular response to toxic substance (GO:0097237)	201	9	1.54	+	5.85	3.36E-05	3.54E-02
autophagy (GO:0006914)	245	10	1.87	+	5.33	2.63E-05	3.45E-02
process utilizing autophagic mechanism (GO:0061919)	245	10	1.87	+	5.33	2.63E-05	3.19E-02
skin development (GO:0043588)	376	12	2.88	+	4.17	4.28E-05	3.56E-02
ncRNA metabolic process (GO:0034660)	537	15	4.11	+	3.65	2.13E-05	3.06E-02
RNA processing (GO:0006396)	850	20	6.5	+	3.08	1.03E-05	2.03E-02
cell death (GO:0008219)	1073	22	8.21	+	2.68	2.90E-05	3.27E-02
programmed cell death (GO:0012501)	1038	21	7.94	+	2.64	5.41E-05	4.06E-02
cellular catabolic process (GO:0044248)	1736	30	13.28	+	2.26	3.56E-05	3.31E-02
catabolic process (GO:0009056)	1972	33	15.09	+	2.19	1.80E-05	3.15E-02
gene expression (GO:0010467)	1986	33	15.2	+	2.17	2.02E-05	3.19E-02
nucleic acid metabolic process (GO:0090304)	2293	35	17.54	+	1.99	6.72E-05	4.82E-02
cellular nitrogen compound metabolic process (GO:0034641)	3575	51	27.35	+	1.86	7.06E-06	1.59E-02
cellular metabolic process (GO:0044237)	7584	92	58.03	+	1.59	8.77E-08	6.92E-04
metabolic process (GO:0008152)	8426	100	64.47	+	1.55	2.48E-08	3.92E-04
nitrogen compound metabolic process (GO:0006807)	7148	83	54.69	+	1.52	5.38E-06	1.41E-02
cellular process (GO:0009987)	14350	136	109.8	+	1.24	3.50E-06	1.10E-02
biological_process (GO:0008150)	17700	155	135.43	+	1.14	1.99E-06	7.84E-03
Unclassified (UNCLASSIFIED)	3342	6	25.57	-	0.23	1.99E-06	1.05E-02

Molecular function

Analysis Type:	PANTHER Overrepresentation Test (Released 20181113)						
Annotation Version and Release Date:	GO Ontology database Released 2018-11-15						
Analyzed List:	upload_1 (Homo sapiens)						
Reference List:	Homo sapiens (all genes in database)						
Test Type:	FISHER						
Correction:	FDR						
GO molecular function complete	Homo sapiens - REFLIST (21042)	upload_1 (161)	upload_1 (expected)	upload_1 (over/under)	upload_1 (fold Enrichment)	upload_1 (raw P-value)	upload_1 (FDR)
RNA binding (GO:0003723)	1670	29	12.78	+	2.27	3.13E-05	3.66E-02
protein binding (GO:0005515)	11870	117	90.82	+	1.29	2.87E-05	4.48E-02
molecular_function (GO:0003674)	17654	154	135.08	+	1.14	6.77E-06	1.58E-02
Unclassified (UNCLASSIFIED)	3388	7	25.92	-	0.27	6.77E-06	3.17E-02

Cellular component

Analysis Type:	PANTHER Overrepresentation Test (Released 20181113)						
Annotation Version and Release Date:	GO Ontology database Released 2018-11-15						
Analyzed List:	upload_1 (Homo sapiens)						
Reference List:	Homo sapiens (all genes in database)						
Test Type:	FISHER						
Correction:	FDR						
GO molecular function complete	Homo sapiens - REFLIST (21042)	upload_1 (161)	upload_1 (expected)	upload_1 (over/under)	upload_1 (fold Enrichment)	upload_1 (raw P-value)	upload_1 (FDR)
RNA binding (GO:0003723)	1670	29	12.78	+	2.27	3.13E-05	3.66E-02
protein binding (GO:0005515)	11870	117	90.82	+	1.29	2.87E-05	4.48E-02
molecular_function (GO:0003674)	17654	154	135.08	+	1.14	6.77E-06	1.58E-02
Unclassified (UNCLASSIFIED)	3388	7	25.92	-	0.27	6.77E-06	3.17E-02

(b) Gene Ontology analysis of 130 proteins down-regulated in organoid skin with EMSY knock-down; results where FDR p<0.05

Biological process and molecular function show no significant enrichments after FDR

Cellular component

Analysis Type:	PANTHER Overrepresentation Test (Released 20181113)						
Annotation Version and Release Date:	GO Ontology database Released 2018-11-15						
Analyzed List:	upload_1 (Homo sapiens)						
Reference List:	Homo sapiens (all genes in database)						
Test Type:	FISHER						
Correction:	FDR						
GO cellular component complete	Homo sapiens - REFLIST (21042)	upload_1 (132)	upload_1 (expected)	upload_1 (over/under)	upload_1 (fold Enrichment)	upload_1 (raw P-value)	upload_1 (FDR)
mitochondrial envelope (GO:0005740)	747	19	4.69	+	4.05	2.89E-07	7.36E-05
mitochondrial membrane (GO:0031966)	705	17	4.42	+	3.84	2.62E-06	4.84E-04
mitochondrial part (GO:0044429)	1042	25	6.54	+	3.82	9.14E-09	4.65E-06
mitochondrial inner membrane (GO:0005743)	472	11	2.96	+	3.72	2.25E-04	2.08E-02
Golgi membrane (GO:0000139)	753	17	4.72	+	3.6	6.15E-06	9.63E-04
Golgi subcompartment (GO:0098791)	856	18	5.37	+	3.35	8.32E-06	1.21E-03
organelle inner membrane (GO:0019866)	532	11	3.34	+	3.3	6.04E-04	4.92E-02
envelope (GO:0031975)	1190	23	7.47	+	3.08	1.63E-06	3.69E-04
organelle envelope (GO:0031967)	1190	23	7.47	+	3.08	1.63E-06	3.32E-04
mitochondrion (GO:0005739)	1595	30	10.01	+	3	5.45E-08	2.22E-05
Golgi apparatus part (GO:0044431)	972	18	6.1	+	2.95	4.36E-05	5.22E-03
Golgi apparatus (GO:0005794)	1564	23	9.81	+	2.34	1.52E-04	1.48E-02
organelle subcompartment (GO:0031984)	1708	25	10.71	+	2.33	7.42E-05	7.95E-03
cytosol (GO:0005829)	5095	66	31.96	+	2.06	2.68E-10	1.82E-07
organelle membrane (GO:0031090)	2978	37	18.68	+	1.98	4.40E-05	4.97E-03
cytoplasmic part (GO:0044444)	9710	107	60.91	+	1.76	3.73E-16	7.59E-13
cytoplasm (GO:0005737)	11571	114	72.59	+	1.57	1.97E-14	2.01E-11
intracellular organelle part (GO:0044446)	9262	85	58.1	+	1.46	3.63E-06	6.16E-04
organelle part (GO:0044422)	9563	85	59.99	+	1.42	1.46E-05	1.98E-03
intracellular membrane-bounded organelle (GO:0043231)	10966	93	68.79	+	1.35	2.30E-05	2.93E-03
intracellular (GO:0005622)	14508	117	91.01	+	1.29	2.00E-07	6.77E-05
intracellular part (GO:0044424)	14508	117	91.01	+	1.29	2.00E-07	5.80E-05
membrane-bounded organelle (GO:0043227)	12474	100	78.25	+	1.28	8.38E-05	8.53E-03
cell part (GO:0044464)	16974	122	106.48	+	1.15	2.33E-04	2.06E-02
cell (GO:0005623)	17037	122	106.88	+	1.14	0.000327	0.0278
