# Supplementary material for: EMSY expression affects multiple components of the skin barrier with relevance to atopic dermatitis
Source: J Allergy Clin Immunol. 2019 Aug;144(2):470–81. doi: 10.1016/j.jaci.2019.05.024 (PMC6683598; doi:10.1016/j.jaci.2019.05.024)

SUPPLEMENTARY FIGURES AND TABLES - continued

EMSY expression affects multiple components of skin barrier with relevance to atopic dermatitis

Martina S Elias PhD,[1]* Sheila C Wright HNC,[1] Judit Remenyi PhD,[1] James C Abbott PhD[2], Susan E Bray PhD[3], Christian Cole PhD[2], Sharon Edwards MBChB[4], Marek Gierlinski PhD[2], Mateusz Glok[1], John A McGrath FRCP[5], William V Nicholson PhD[1], Lavinia Paternoster PhD[6], Alan R Prescott PhD[7], Sara Ten Have PhD[8], Phillip D Whitfield PhD[9], Angus I Lamond PhD[8] and Sara J Brown FRCPE[1,10]*

Fig E8. qPCR, Western blotting and immunofluorescence to test for validation of selected protein expression changes identified by mass spec analysis

Gene	Protein	Mean FC in m/s proteomics (n=4)	Function	qPCR (fold change n=4, mean ± SEM)	Western blotting (representative images from ≥4 bio-replicates)	Immunostaining (representative images from ≥3 bio-replicates)
EMSY	EMSY	Below threshold for detection in mass spec analysis	Transcriptional regulator	0.68 ± 0.04	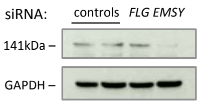	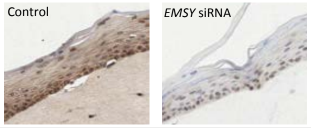
FLG	Filaggrin	12.5	Structural and multifunctional	5.07 ± 4.78	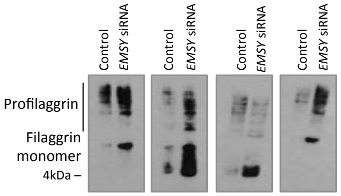	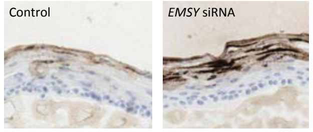
FLG2	Filaggrin 2	38.1		25.28 ± 19.57	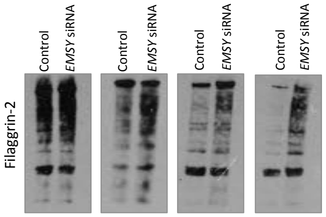	Not done
KRT2	Keratin 2	7.9		41.90 ± 34.94	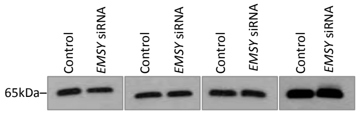	Immunofluorescence images shown following this table
HAL	Histidine ammonia lyase	11.9	Filaggrin processing	17.92 ± 14.23	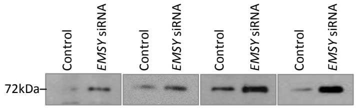	Immunofluorescence images shown following this table
BLMH	Bleomycin hydrolase	5.8		8.50 ± 2.78	Not done	Not done
ASPRV1	Retroviral-like aspartic protease 1	11.8		43.92 ± 36.25	Not done	Not done

Gene	Protein	Mean FC in m/s proteomics (n=4)	Function	qPCR (fold change n=4, mean ± SEM)	Western blotting (representative images from ≥4 bio-replicates)	Immunofluorescence (representative images from ≥3 bio-replicates)
STS	Steroid sulphatase	7.3	Lipid processing and metabolism	1.25 ± 0.45	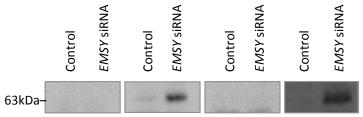	Nonspecific staining
ALOXE3	Arachidonate lipoxygenase 3	9.7		4.84 ± 1.25	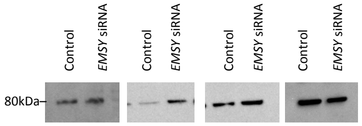	Nonspecific staining
ALOX12B	Arachidonate lipoxygenase 12	7.4		5.43 ± 2.61	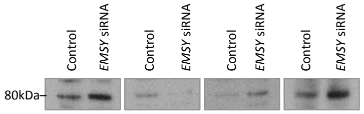	Immunofluorescence images shown following this table
APOE	Apolipoprotein E	3.1		1.87 ± 0.36	Not done	Not done
CDSN	Corneodesmosin	51.6	Cell-cell adhesion	56.38 ± 50.78	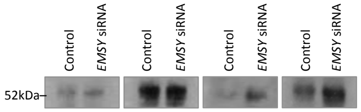	Immunofluorescence images shown following this table
DSC1	Desmocollin 1	7.2		2.90 ± 0.47	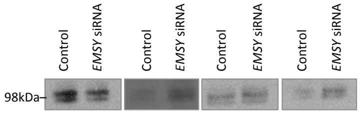	Immunofluorescence images shown following this table
GJA1	Gap junction alpha-1 protein (connexin 43)	2.5	Cell-cell communication	1.21 ± 0.27	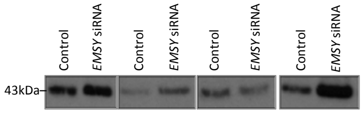	Nonspecific staining

Gene	Protein	Mean FC in m/s proteomics (n=4)	Function	qPCR (fold change n=4, mean ± SEM)	Western blotting (representative images from ≥4 bio-replicates)	Immunofluorescence (representative images from ≥3 bio-replicates)
IL36RN	Interleukin-36 receptor antagonist protein	4.2	Control of inflammation	6.70 ± 4.03	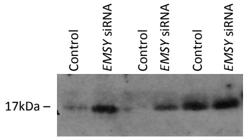	Nonspecific staining
mTOR	Mechanistic target of rapamycin	0.3	Cell cycle and cell proliferation	1.36 ± 0.25	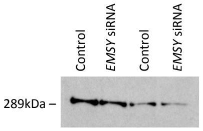	Immunofluorescence images shown following this table
CDK1	Cyclin-dependent kinase 1	6.8	Cell cycle	1.32 ± 0.21	Not done	Not done
COL7A1	Collagen alpha-1 7 chain	5.3	Basement membrane	1.46 ± 0.53	Not done	No clear staining seen
RNAse7	Ribonuclease 7	20.5	Antimicrobial	17.76 ± 15.13	Not done	Not done

Keratin 2 DAPI
Composite image created by Zeiss LSM710 microscope

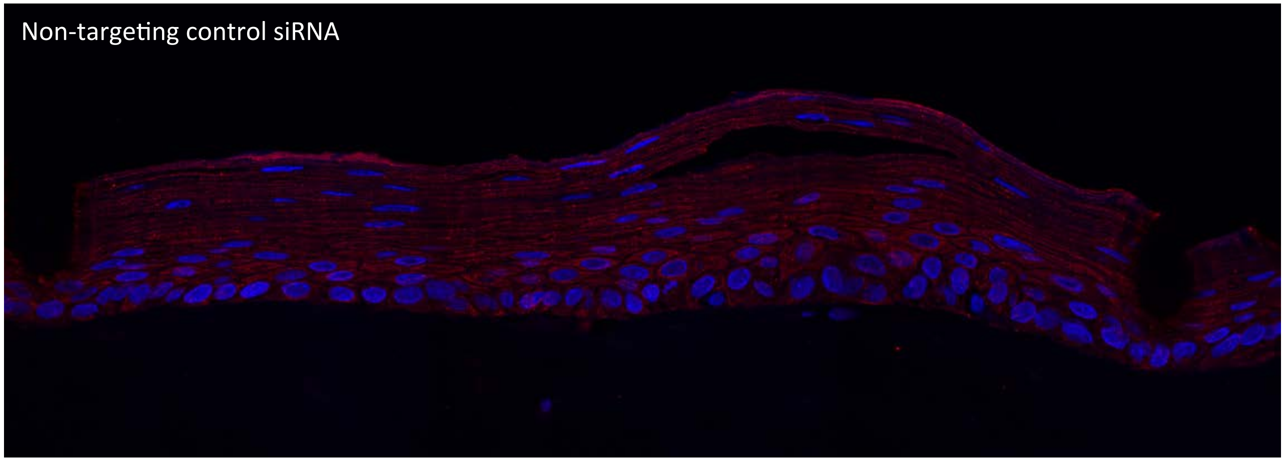

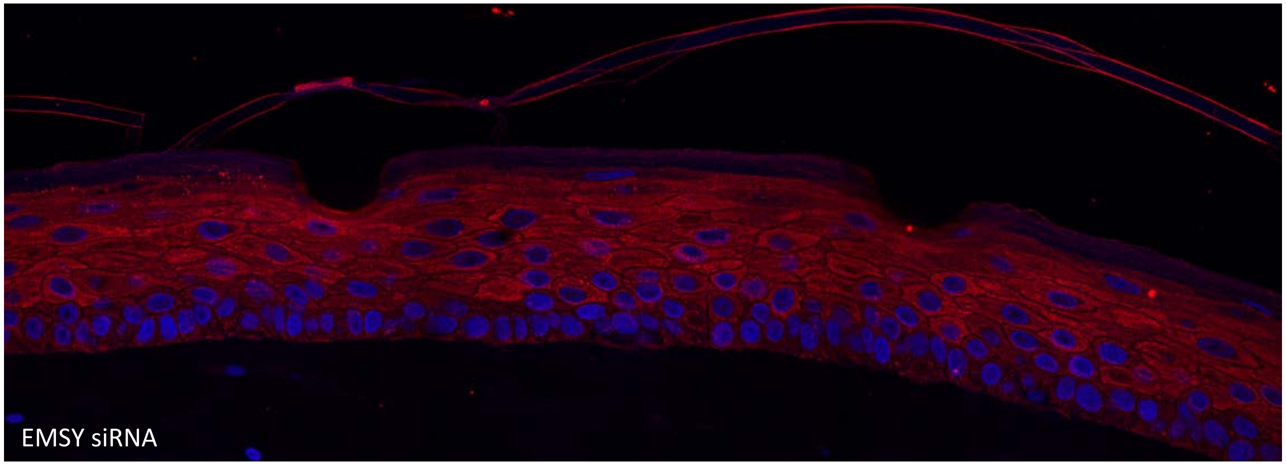

Histidine ammonia lyase DAPI

Composite image created by Zeiss LSM710 microscope

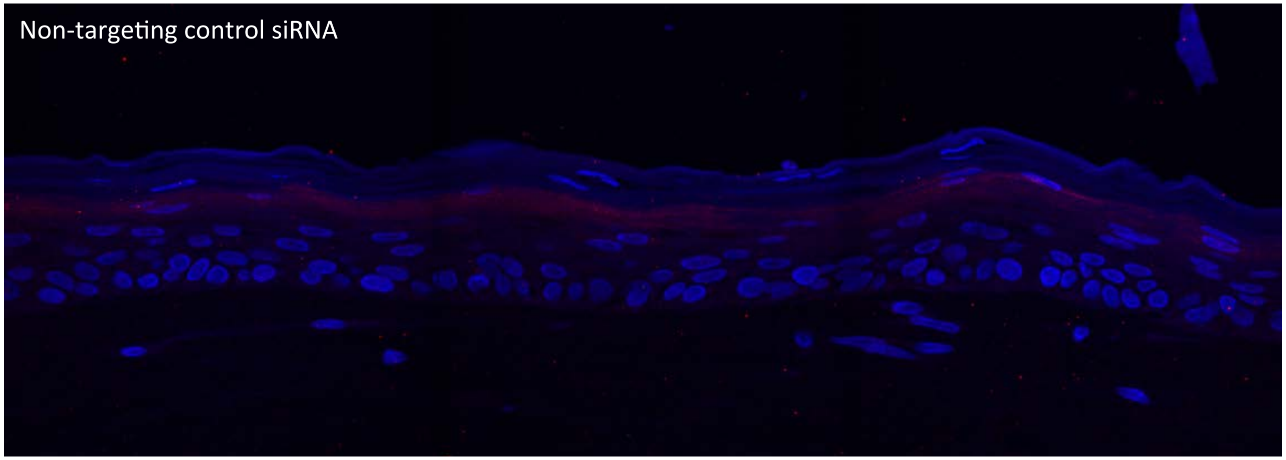

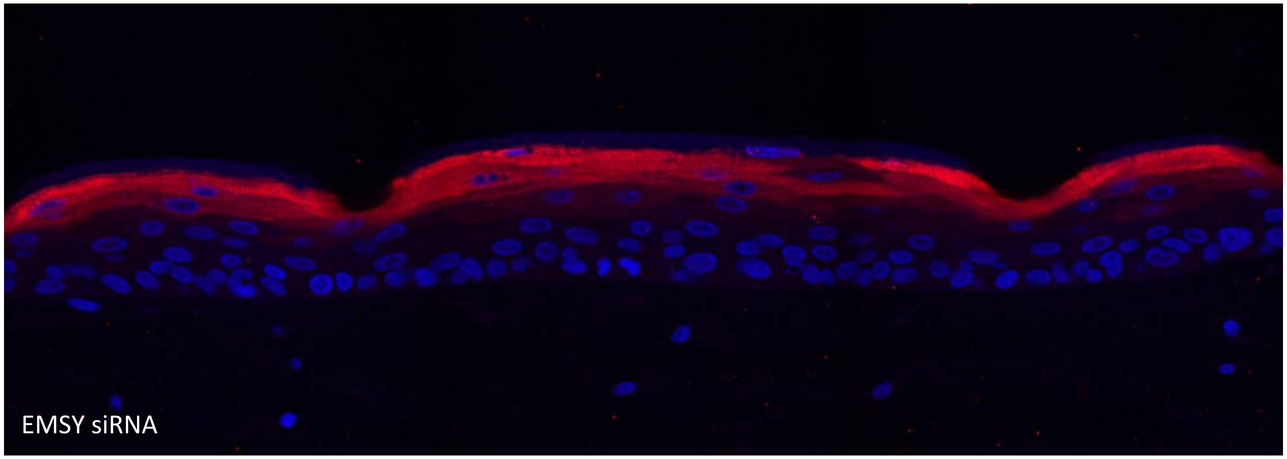

ALOX12 DAPI

Composite image created by Zeiss LSM710 microscope

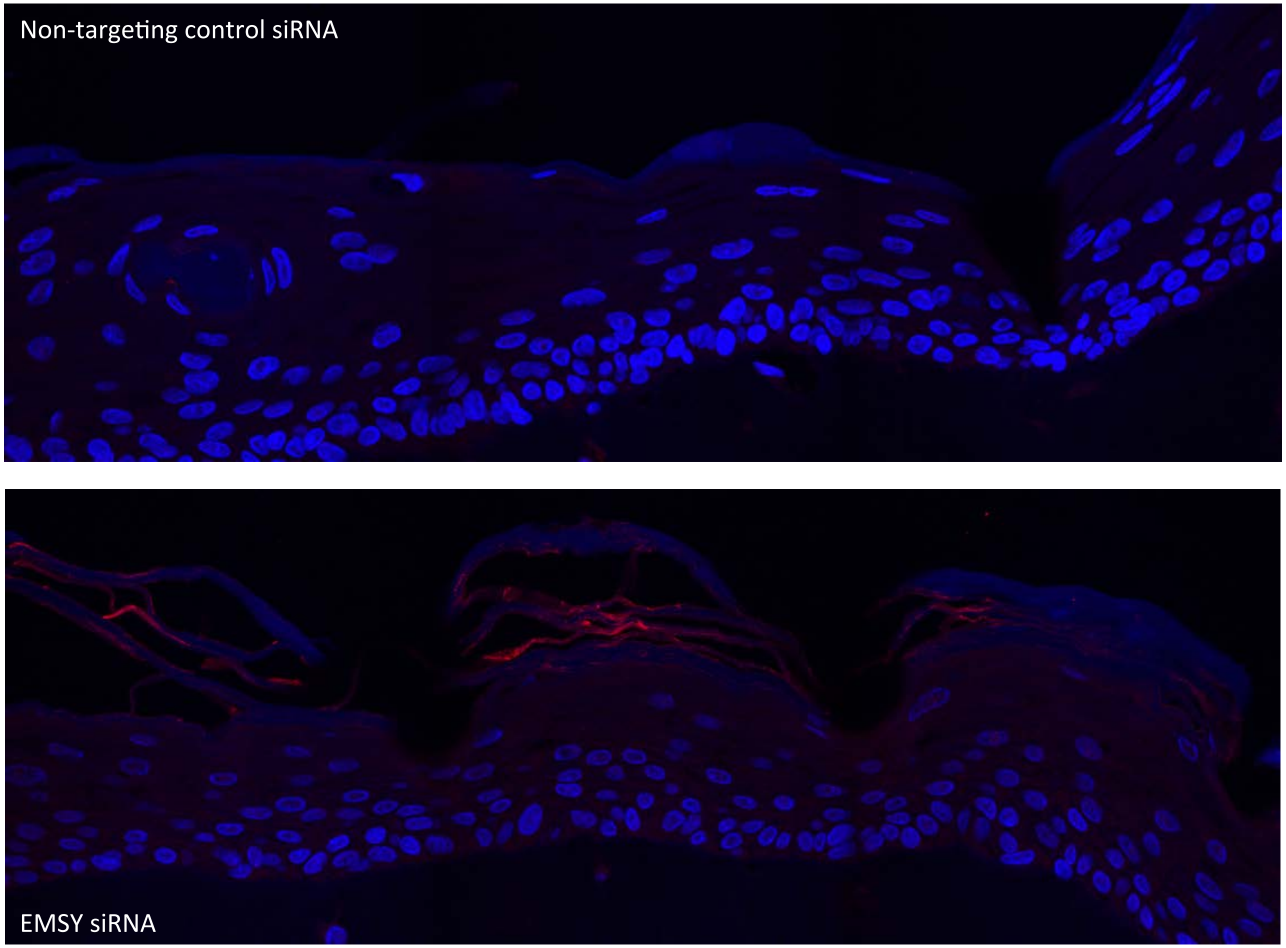

Corneodesmosin DAPI
Composite image created by Zeiss LSM710 microscope

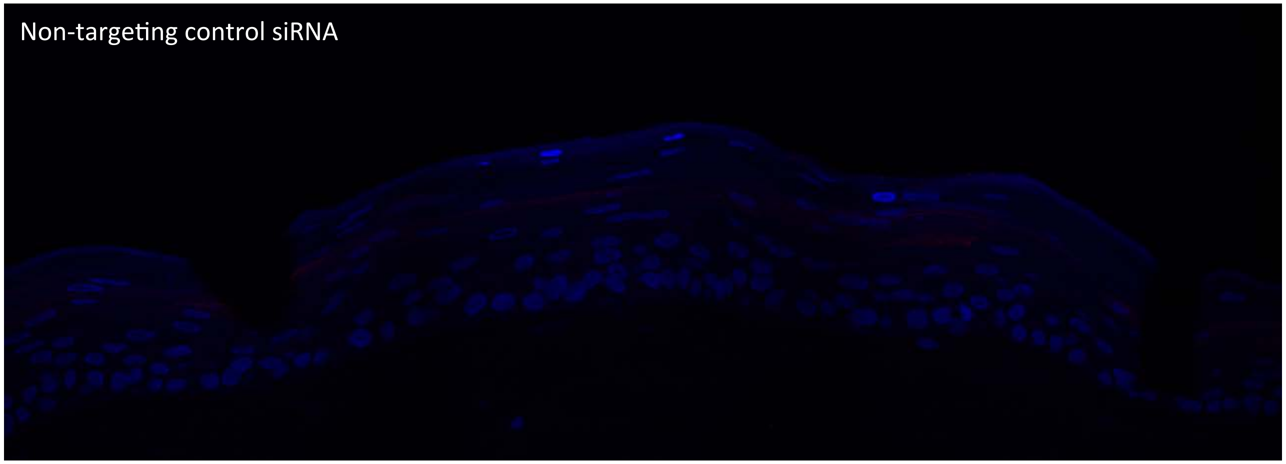

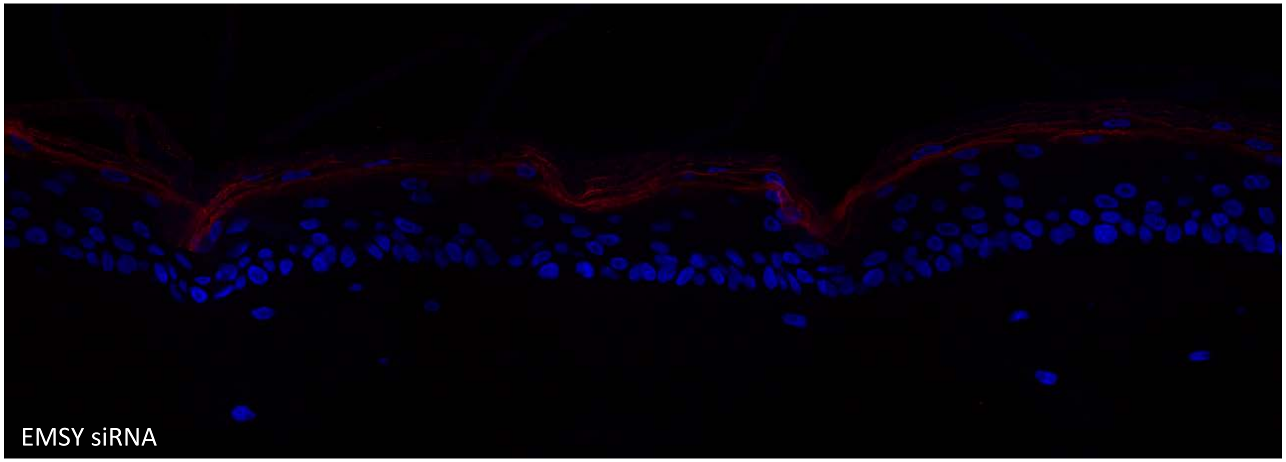

Desmocollin DAPI
Composite image created by Zeiss LSM710 microscope

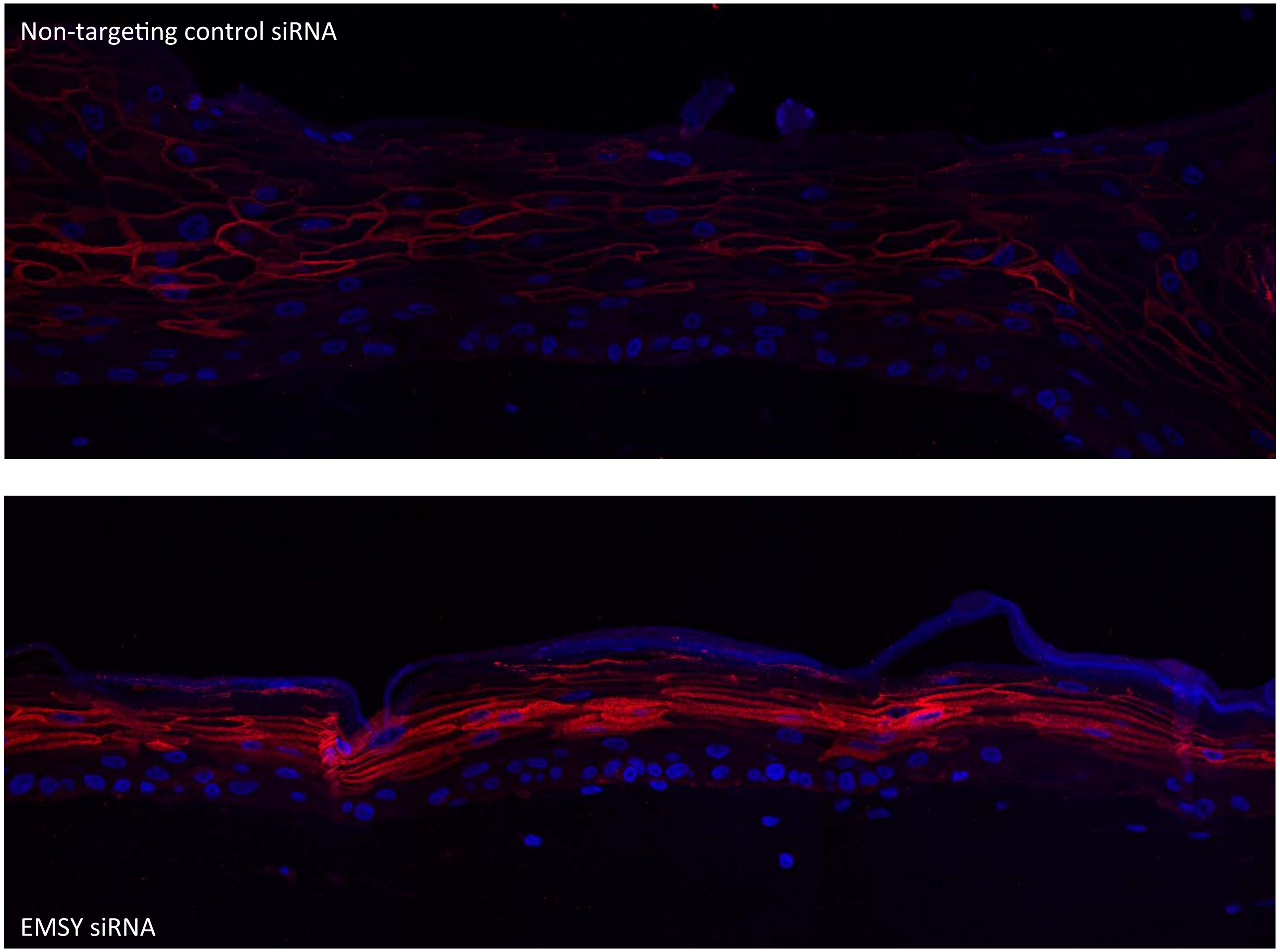

mTOR DAPI

Composite image created by Zeiss LSM710 microscope

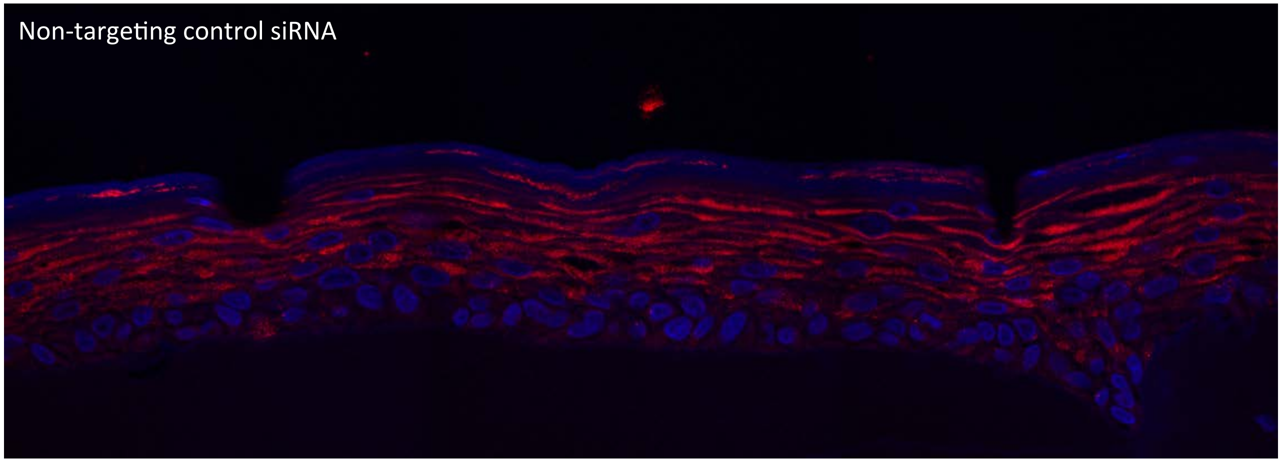

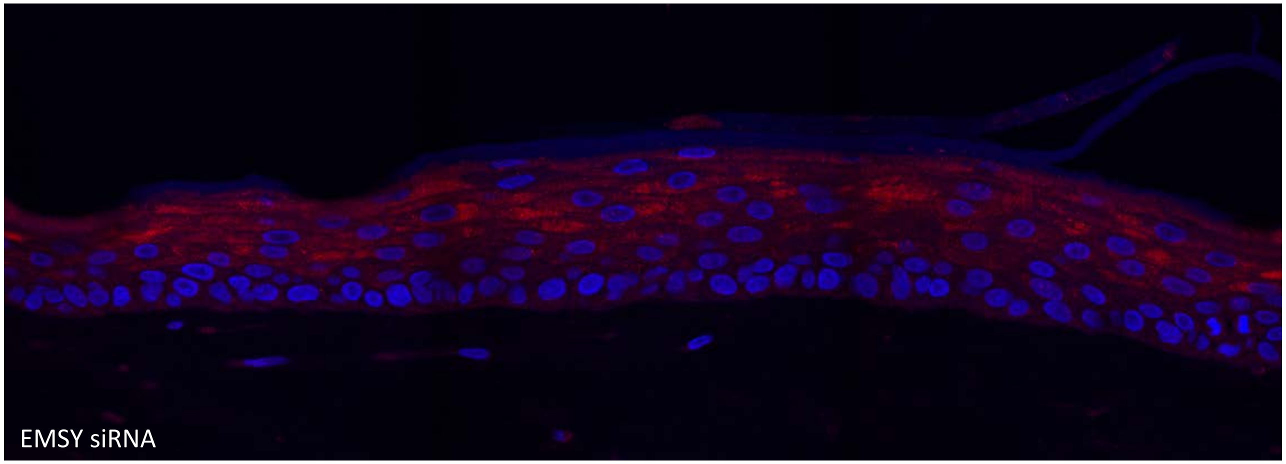

Supplement: Fig E8 [file mmc3.pdf]
