# Supplementary material for: EMSY expression affects multiple components of the skin barrier with relevance to atopic dermatitis
Source: J Allergy Clin Immunol. 2019 Aug;144(2):470–81. doi: 10.1016/j.jaci.2019.05.024 (PMC6683598; doi:10.1016/j.jaci.2019.05.024)

SUPPLEMENTARY FIGURES AND TABLES – continued

EMSY expression affects multiple components of skin barrier with relevance to atopic dermatitis

Martina S Elias PhD,[1]* Sheila C Wright HNC,[1] Judit Remenyi PhD,[1] James C Abbott PhD[2], Susan E Bray PhD[3], Christian Cole PhD[2], Sharon Edwards MBChB[4], Marek Gierlinski PhD[2], Mateusz Glok[1], John A McGrath FRCP[5], William V Nicholson PhD[1], Lavinia Paternoster PhD[6], Alan R Prescott PhD[7], Sara Ten Have PhD[8], Phillip D Whitfield PhD[9], Angus I Lamond PhD[8] and Sara J Brown FRCPE[1,10]*

Fig E9. Transmission electron microscopy images of organoid skin model with osmium staining, showing ultrastructure of stratum corneum

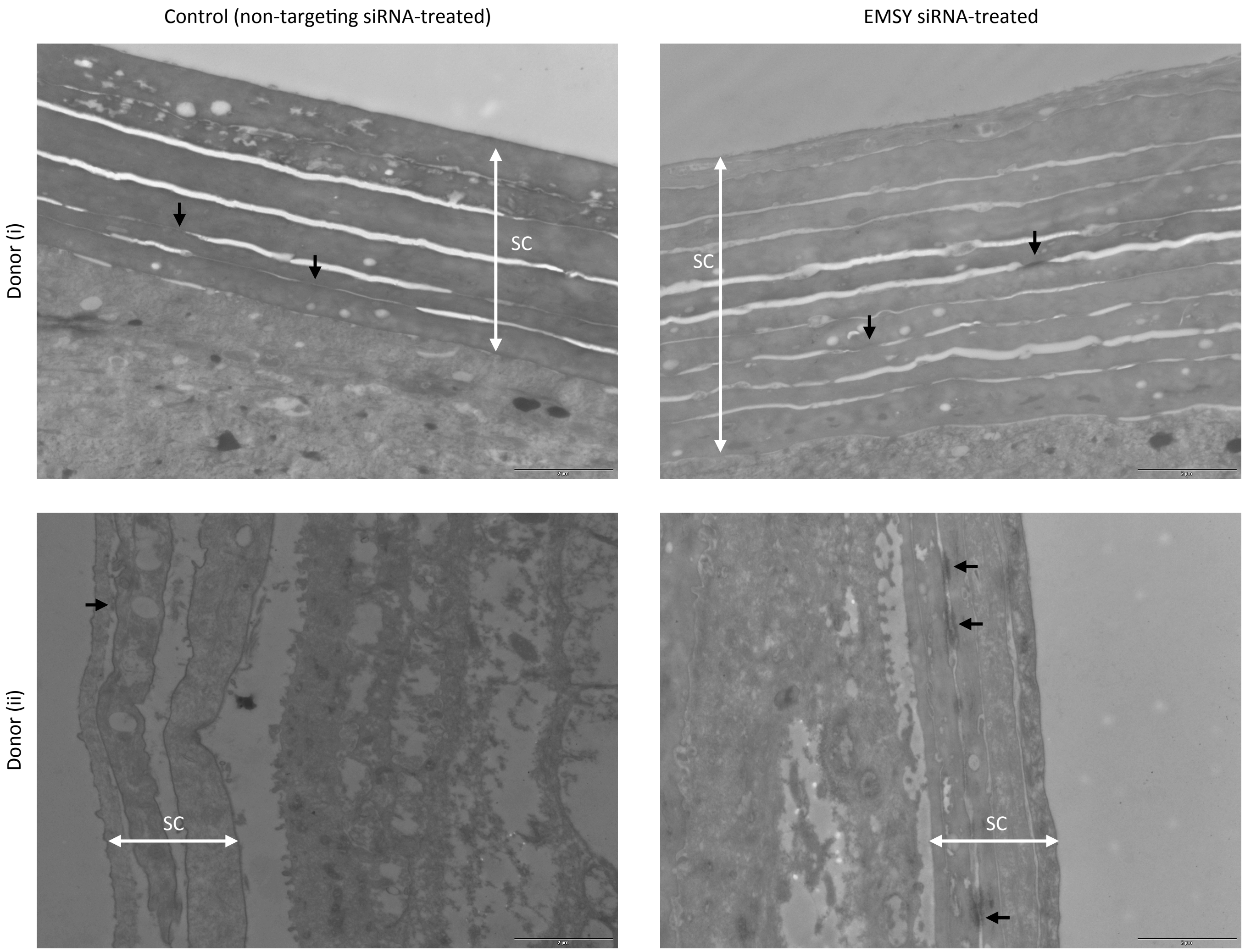

[Note: These upper two Images are reproduced here from Figure 2F, to allow comparison with replicate experiment.]

EMSY knock-down produces a thicker stratum corneum (SC) with an increased number of component layers, consistent with the increase in corneodesmosin detected by mass spectrometry; black arrows indicate corneodesmosomes; representative images from four biological replicate experiments.

Fig E10. Immunohistochemistry to investigate EMSY expression in skin

Normal control skin obtained from plastic surgical excision of breast or abdominal skin

Dermatitis cases with clinical and/or histological features of AD from different body sites as labelled

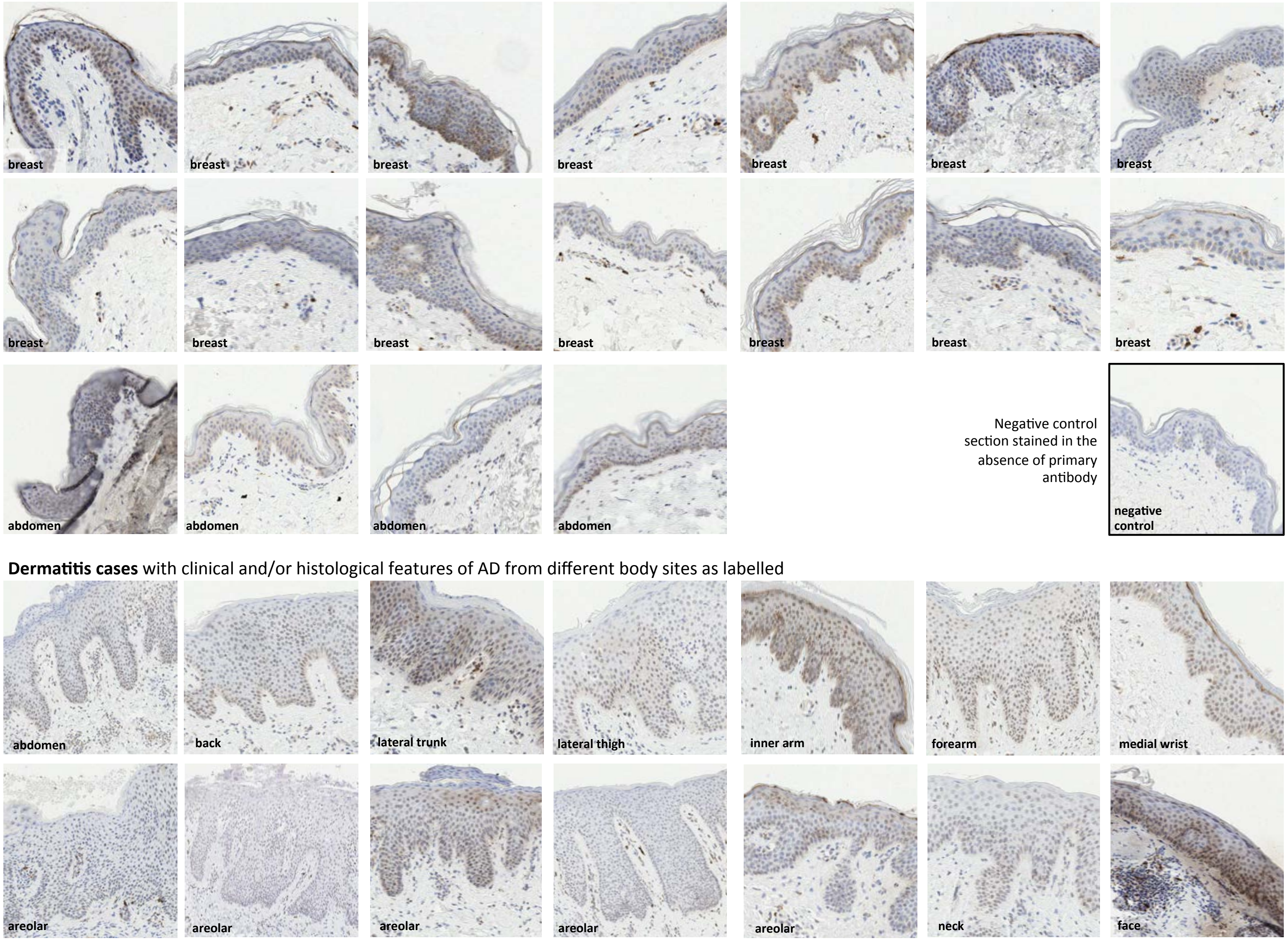

Supplement: Figs E9 and E10 [file mmc4.pdf]
